# The disadvantages of being a hybrid during drought: A combined analysis of plant morphology, physiology and leaf proteome in maize

**DOI:** 10.1371/journal.pone.0176121

**Published:** 2017-04-18

**Authors:** Dana Holá, Monika Benešová, Lukáš Fischer, Daniel Haisel, František Hnilička, Helena Hniličková, Petr L. Jedelský, Marie Kočová, Dagmar Procházková, Olga Rothová, Lenka Tůmová, Naďa Wilhelmová

**Affiliations:** 1 Department of Genetics and Microbiology, Faculty of Science, Charles University, Prague, Czech Republic; 2 Department of Experimental Plant Biology, Faculty of Science, Charles University, Prague, Czech Republic; 3 Institute of Experimental Botany, Academy of Sciences of the Czech Republic, Prague, Czech Republic; 4 Department of Botany and Plant Physiology, Faculty of Agrobiology, Food and Natural Resources, Czech University of Life Sciences Prague, Prague, Czech Republic; 5 Department of Cell Biology, Faculty of Science, Charles University, Prague, Czech Republic; Estacion Experimental del Zaidin, SPAIN

## Abstract

A comparative analysis of various parameters that characterize plant morphology, growth, water status, photosynthesis, cell damage, and antioxidative and osmoprotective systems together with an iTRAQ analysis of the leaf proteome was performed in two inbred lines of maize (*Zea mays* L.) differing in drought susceptibility and their reciprocal F1 hybrids. The aim of this study was to dissect the parent-hybrid relationships to better understand the mechanisms of the heterotic effect and its potential association with the stress response. The results clearly showed that the four examined genotypes have completely different strategies for coping with limited water availability and that the inherent properties of the F1 hybrids, *i*.*e*. positive heterosis in morphological parameters (or, more generally, a larger plant body) becomes a distinct disadvantage when the water supply is limited. However, although a greater loss of photosynthetic efficiency was an inherent disadvantage, the precise causes and consequences of the original predisposition towards faster growth and biomass accumulation differed even between reciprocal hybrids. Both maternal and paternal parents could be imitated by their progeny in some aspects of the drought response (*e*.*g*., the absence of general protein down-regulation, changes in the levels of some carbon fixation or other photosynthetic proteins). Nevertheless, other features (*e*.*g*., dehydrin or light-harvesting protein contents, reduced chloroplast proteosynthesis) were quite unique to a particular hybrid. Our study also confirmed that the strategy for leaving stomata open even when the water supply is limited (coupled to a smaller body size and some other physiological properties), observed in one of our inbred lines, is associated with drought-resistance not only during mild drought (as we showed previously) but also during more severe drought conditions.

## Introduction

Plant hybrids often display superiority to their parents in terms of various morphological and physiological traits. This phenomenon is usually referred to as “heterosis” or “hybrid vigour”, although these two terms are not completely interchangeable and heterosis does not have to be only positive [1]. The scientific investigation of heterosis in plants dates to the 18^th^ and 19^th^ centuries and the first attempts to explain heterosis at a genetic level appeared soon after the re-discovery of Mendel´s principles of inheritance (reviewed *e*.*g*. by [2]). The advent of molecular biology and particularly the development of high-throughput “omics” technologies provided interesting information on the possible molecular basis of heterosis. Hybrids are usually characterized by different levels of transcripts compared to their parents and although simple additivity seems to prevail (at least in most cases), a considerable number of genes have been shown to have an allele-specific type of expression that could certainly result in heterosis [3–4]. This can be associated with an allele-specific regulation of gene expression by small RNAs [1], the presence of epigenetic marks on chromatin (DNA methylation, histone modifications) [1–3] or an allele-specific binding of transcriptional factors [2]. However, as most of this information was obtained from analyses of a transcriptome (or, more recently, an epigenome), it is necessary to realize that the observed parent/hybrid differences in the regulation of transcription do not have to be reflected in corresponding differences in levels/activities of various proteins. In fact, when transcriptome and proteome were analysed jointly in maize roots, the percentage of proteins accumulating non-additively in a hybrid quite substantially differed from the percentage of transcripts with a non-additive pattern (49% *vs* 18%, respectively [5]).

The number of studies dealing with an analysis of heterosis in plants at the proteome level is still rather small (recently reviewed by [6]). Most of this work was performed with maize, which is understandable because this species is the best known and the most agronomically important example of a manifestation of heterosis in plants. Several authors [7–9] analysed a differential accumulation of proteins in embryos of maize F1 hybrids and their inbred parents. Proteomic and metabolomic analyses of maize seeds to examine possible molecular mechanisms of heterosis were performed by [10]. The proteome of coleoptiles and plumules in maize hybrids and inbreds was examined by [11], the total and mitochondrial proteome of ear shoots by [12–13] and the proteome of primary or seminal roots by [5, 14–15]. Only one study [16] dealt with molecular aspects of heterosis at the level of the leaf proteome. Regarding the leaf proteome of other plant species, some work has been conducted in rice [17], wheat [18–19], sunflower [20–21], soybean [22] and sorghum-sudangrass hybrids [23]. Most these authors found a non-additive pattern of expression for at least some proteins (the percentage varied between 10 and 80% of all detected proteins depending on the respective study) in their hybrid experimental material. Qualitative differences and isoform variation between hybrids and their respective parents were also observed in some cases. Generally, proteins that show heterotic patterns belong mostly to the categories of cell metabolism (including photosynthesis, carbon and energy metabolism, amino acid and protein metabolism and secondary metabolism), cell division and growth, cell detoxification, stress response, defence and disease-related pathways, signal transduction and the regulation of gene expression.

The results of proteomic, transcriptomic and epigenomic studies, together with the results of some metabolome analyses and with data obtained by examination of some biochemical and physiological parameters in heterotic hybrids, has led some scientists to propose various heterosis models. These models attempt to explain how changes in the expression of genes belonging to some specific functional category (both at RNA and protein levels) induce changes in cell metabolism that eventually result in a manifestation of heterosis on a whole plant level (reviewed *e*.*g*. by [3]). Of particular interest is the model proposing an increased carbon gain/energy input in hybrids due to their increased photosynthetic efficiency [2–3, 24]. This could be associated with an altered regulation of circadian clock genes [25–26], which affects not only photosynthesis but also participates in a general plant stress response [1]. A role for reduced expression of defence and stress-associated genes coupled to decreased levels of salicylic acid and increased levels of auxin in heterotic hybrids was recently proposed by [27]. These authors argue that such changes would enable increased growth of hybrids because plant immunity and growth processes are antagonistic. Another interesting possibility is the model of Goff [28] which proposes that hybrids more efficiently use their available sources of energy in comparison to their inbred parents due to a reduction in their protein metabolism processes and greater protein stability. This could also give them an advantage over a larger scale of environmental conditions [4].

The environmental conditions play an important role throughout plant life. A better ability of hybrids to maintain cell homeostasis and full metabolic functionality even in the presence of some abiotic or biotic stressor would certainly be very advantageous. This should manifest as an increase in heterosis in stressed plants in comparison to non-stressed ones. Such a phenomenon has been documented by numerous experimental data; however, these data usually deal only with yield-associated traits or general plant morphology. An analysis of the physiology and biochemistry of stressed plants in relation to heterosis is less frequent and gene expression analyses of hybrid/parent differences under such conditions are even rarer. Abraham Blum in his excellent paper on this topic reviewed several possible physiological mechanisms that could explain, *e*.*g*., the increase in heterosis observed in plants subjected to sub-optimal or supraoptimal temperature or high irradiance conditions [24]. For these types of stressors, the hybrid superiority observed on a whole plant level is usually accompanied/caused by a similar hybrid superiority in efficiency of thylakoid photosynthetic complexes [29–32], the content of photosynthetic pigments [33] or soluble sugars [34–35], the protective capacity of antioxidative systems [31–32, 36], *etc*.

However, the features that are applicable to one type of stress, do not necessarily apply to others. For example, heterosis in plants subjected to water deficit is rather ambiguous. Drought has sometimes been documented to increase heterosis in yield traits (*e*.*g*. [37–38]), but in other cases, a drought-induced decrease of the heterotic effect in morphological/yield parameters was observed [39–41]. Studies analysing differences between hybrids and their parents in drought-stressed plants at a molecular level are extremely rare. Drought-induced changes in allele-specific expression of protein-coding genes at a transcriptome level were analysed in maize, barley and rice by [42–45]. Other authors examined the differential expression of some miRNAs in maize subjected to water stress [46], methylation levels in drought-susceptible and -tolerant rice parents and their F1 hybrids [47] and parent/hybrid differences in the root proteome of rapeseed under drought stress [48]. To our knowledge, a really complex study of heterosis and its association with possible drought resistance, which would combine molecular, biochemical, physiological and morphological approaches together, does not exist.

Thus, we have decided to perform such a study and to examine at various levels whether hybrids can really be better adapted to drought conditions than their inbred parents. This paper presents the results of a thorough examination of the leaf proteome, various aspects of photosynthesis, plant water management and cell protective processes together with an assessment of plant morphology, development and biomass production under optimum or insufficient water supply conditions. This analysis was performed in the young maize plants of two inbred lines that differ in drought resistance and their reciprocal hybrids of F1 generation.

## Materials and methods

### Plant material and cultivation conditions

Two inbred lines of maize (*Zea mays* L.), the drought-sensitive 2023 and the drought-resistant CE704, were used as the experimental material together with their reciprocal F1 hybrids 2023×CE704 and CE704×2023. All genotypes originated from the breeding programme of the *CEZEA Maize Breeding Station* (Čejč, Czech Republic). The evaluation of inbred lines for their drought susceptibility was based on the analysis of shoot biomass data collected from a genotypic set of 30 inbred lines evaluated under the same conditions as those used for this study. The CE704 ranked the best with values of stress susceptibility index (SSI) of 0.52 and stress tolerance index (TOL) of 0.24, and 2023 ranked the worst with an SSI value of 1.24 and a TOL value of 1.90; SSI and TOL indices were calculated according to [49] and [50], respectively.

Plants were cultivated in pots (12 cm diameter, 13 cm depth, one plant per pot) filled with a mixture (2:1 v/v) of garden soil substrate (Agro CS) and sand (soil: 120 mg L^-1^ N, 100 mg L^-1^ P_2_O_5_, 150 mg L^-1^ K_2_O, pH 5.5–6, sand: 99.67% SiO_2_, 0.13% Al_2_O_2_, 0.06% Fe_2_O_3_, 0.12% TiO_2_, 0.02% CaO) and placed in a naturally-lit greenhouse under semi-controlled conditions (air temperature 25±2/20±2°C, relative air humidity 50±5/70±5% day/night). Plants were sufficiently watered until 35 days after the date of sowing, when they were divided into two groups. The first group (control) continued to be sufficiently watered (*i*.*e*., twice daily, to maintain the volumetric soil water content at the level of approx. 25–30%) for the next 10 days, whereas complete cessation of watering in the second (stressed) group of plants resulted in a simulation of drought conditions (volumetric soil water content of approx. 1%) (S1 File). At the start of the drought simulation, all plants had 3–4 fully developed and completely green leaves. The experiments were conducted in two independent series with a completely randomized design; each variant (genotype/water treatment combination) in each series was represented by 90 plants. At the end of the drought simulation period, all measurements and necessary samplings were conducted (the 5^th^ leaf, which was fully developed in all plants at this time, was always utilized). Whole plants or other plant parts were also utilized for the evaluation of plant morphology.

### Evaluation of plant morphology

The number of fully developed leaves and the height of plants (measured as the distance from the soil level in pots to the tip of the youngest leaf visible in the top whorl of leaves) were determined in twenty plants of each genotype/water treatment combination. Fresh (FM) and dry (DM) masses of the shoot and roots of the same plants were recorded. The same plants also served for the assessment of the total area of photosynthetically active leaves (LA), which was based on the calculations of the area of individual leaves (only leaves with green colour visible for at least two thirds of their length were included). Leaf area ratio (LAR) was calculated as the ratio of LA and DM of photosynthetically active leaves.

### Plant water status and osmotic potential determination

The relative water content (RWC) in leaves was calculated as 100×(LFM–LDM)/(LSM–LDM), where LFM represents the fresh mass of 10 leaf discs (diameter 0.6 cm) cut from the middle portion of the leaf blade and immediately weighed, LSM is the saturated mass of the same discs after their hydration in the dark for 5 h, and LDM is the dry mass of these discs after they were oven-dried at 80°C for 24 h. Ten plants were evaluated for each genotype/water treatment combination.

The leaf osmotic potential (OP) was measured using the psychrometer *PSYPRO* with Wescor´s sample chamber, model C-52 (*Wescor Inc*., Logan, UT, USA). Samples (6 g) consisting of three leaf segments collected from the leaves of five plants of each variant were collected between 8:00 and 9:00, Central European time (CET) and kept gently compressed in insulin syringes sealed with Para film at -18°C. Prior to actual measurements, the syringes were left at 2°C until the tissue was completely thawed. A few drops of the cell sap from each syringe were then pushed out into the measuring chamber so that the bottom of the measuring tray in the measuring chamber was entirely covered with the cell sap. Each measurement was carried out for a period of 60 min.

### Leaf gas exchange measurements

The portable gas exchange system *LCpro+* (*ADC BioScientific*, Hoddesdon, Great Britain) was used for *in situ* determination of the net photosynthetic rate (P_N_), the rate of transpiration (E), and the stomatal conductance (g_S_). All measurements were conducted between 8:00 and 11:30, CET. The duration of each individual measurement was 10 min after the establishment of steady-state conditions inside the measurement chamber. The conditions in the chamber were as follows: temperature 25°C, ambient CO_2_ concentration 550±50 μL L^-1^, air flow rate 205±30 μmol s^-1^ and irradiance 650±50 μmol m^-2^ s^-1^ of photosynthetically active radiation. The intrinsic water use efficiency (WUE) was calculated as the ratio of P_N_/E. Each genotype/water treatment combination was represented by 20–30 individual plants.

### Determination of photosynthetic pigments content

Six independent samples were prepared from 10–15 leaves for each genotype/water treatment combination. The sampling occurred between 10:30 and 11:00, CET. Leaf discs (diameter 0.4–0.8 cm) were cut from the middle portion of the leaf blade. The content of individual photosynthetic pigments (chlorophylls and carotenoids) was determined in acetone extracts generated from these samples. The analysis was performed using HPLC with a reverse-phase column (*Watrex Nucleosil 120 5 C18*, 5 μm particle size, 125×4 mm, *ECOM*, Prague, Czech Republic). The solvent system was acetonitrile/methanol/water (80:12:10 v/v/v) followed by methanol/ethyl acetate (95:5 v/v), and the linear gradient was run from 2 to 6 min (flow rate 1 mL min^-1^, detection wavelength 445 nm). Data were captured and calculated using *Clarity* software (*DataApex*, Prague, Czech Republic). The deepoxidation state of xanthophyll cycle pigments (DEPS) was calculated from the contents of zeaxanthin (Z), antheraxanthin (A) and violaxanthin (V) as DEPS = [(Z+0.5×A)/(Z+A+V)].

### Evaluation of primary photosynthetic processes

Chlorophyll (Chl) fluorescence measurements and leaf samplings for the isolation of chloroplasts occured between 8:00 and 9:00, CET. The measurements of the polyphasic rise of Chl fluorescence transient (O-J-I-P) were performed on the upper surface of dark-adapted (20 min) leaves (the middle portion of the leaf blade) *in situ* with the portable fluorometer *FluorPen FP100max* (*Photon System Instruments*, Brno, Czech Republic). The intensity of the saturating pulse (blue light, 455 nm) was 3000 μmol m^-2^ s^-1^. All Chl fluorescence transients were recorded with a time scan from 10 μs to 2 ms. Fluorescence values recorded at 50 μs (F_0_, initial fluorescence intensity), 300 μs (F_K_, fluorescence intensity at the K-step), 2 ms (F_J_, fluorescence intensity at the J-step), 60 ms (F_I_, fluorescence intensity at the I-step), and F_M_ ≈ F_P_ (maximum fluorescence intensity) were used to calculate various parameters of the JIP test based on the theory of energy flow in the photosynthetic electron-transport chain according to [51] and [52]. Calculations of the relative variable fluorescence (*i*.*e*., normalization of whole fluorescence transients) and the difference kinetics were used to obtain further information regarding the primary photosynthetic processes as described by [53]. Twenty individual plants were assessed per genotype/water treatment combination.

The photochemically active broken mesophyll chloroplasts were isolated from 1.5–2 g of leaf tissue using the procedure described by [36]. The resulting chloroplast suspensions were maintained at 0°C and in the dark until the measurements of Photosystem (PS) I and II activities. These activities were evaluated polarographically using a Clark type oxygen electrode (*Theta ´90*, Prague, Czech Republic) as the amount of oxygen formed (PSII) or consumed (PSI) by the chloroplast suspensions after their irradiation with white light (850 μmol m^-2^ s^-1^) and the addition of artificial electron acceptors and donors. The measurement chamber was constructed as described by [54], and the details of the measurements are described in [30]. The only modifications from the procedure described in [30] were the utilization of 2 mM potassium ferricyanide together with 1 mM 2,6-dimethylbenzoquinone as artificial electron acceptors for the measurement of PSII activity, and the addition of 5 mM NaN_3_ (final concentration in the measurement chamber) to ensure an inhibition of the activity of endogenous catalases during the measurements of PSI activity. Four independent samples, each prepared from eight leaves, represented each genotype/water treatment combination and both PSI and PSII activities were measured 2 to 4 times in each sample.

### Cell membrane injury determination

The cell membrane injury (MI) was determined as described by [55]. Sixty leaf discs (0.5 cm diameter) were cut from the middle portion of the leaf blade (five plants per variant) and washed out several times with distilled water. The sampling occurred between 8:00 and 9:00, CET. One half of the discs (T) was placed in 10 mL of 30% polyethylene glycol 6000 and kept at 8°C for 24 h, then washed out several times with distilled water and kept in 30 mL of distilled water at 8°C for 24 h; the other half (C) was subjected only to the treatment with distilled water. Both types of samples were then warmed at 25°C, and their electrical conductivity (T_1_, C_1_) was measured using the *GRYF 158* conductometer (*Gryf HB*, Havlíčkův Brod, Czech Republic). Samples were then boiled for 15 min and the electrical conductivity was again measured (T_2_, C_2_). MI was calculated as 100-MS, where MS = 100×[(1-T_1_/T_2_)/(1-C_1_/C_2_)].

### Determination of the activities/contents of antioxidants

The sampling occurred between 10:30 and 11:00, CET, and the samples were frozen in liquid nitrogen and stored at -70°C until determination of the activities of antioxidative enzymes or the contents of ascorbate, glutathione and proline. Soluble protein extracts were prepared as described by [56]. The activities of ascorbate peroxidase (APX, E.C. 1.11.1.11), glutathione reductase (GR, EC 1.6.4.2) and superoxide dismutase (SOD, EC 1.15.1.1) were measured spectrophotometrically (*Hitachi U 3300*, *Hitachi High-Tech Corporation*, Tokyo, Japan) at 25°C. The activity of APX was determined by the decrease in reduced ascorbate content at 290 nm as described by [57]. The GR activity was assayed as described by [58] by the increase in absorbance at 412 nm due to the formation of a coloured complex of reduced glutathione, produced by GR, with 5-(3-carboxy-4-nitrophenyl)disulfanyl-2-nitrobenzoic acid. SOD activity was measured at 470 nm; the production of superoxide was provided by the conversion of xanthine catalysed by xanthine oxidase [59]. One unit of SOD activity was defined as the amount of enzyme required for 50% inhibition of the reaction rate of 2,3-bis(2-methoxy-4-nitro-5-sulfophenyl)-2H-tetrazolium-5-carboxanilide inner salt, a detection molecule that is reduced by superoxide. The activity of catalase (CAT, EC 1.11.1.6) was measured polarographically using an oxygen electrode (*Hansatech Instruments*, King´s Lynn, Great Britain) as described by [60]. The protein content was determined spectrophotometrically using the Bradford assay [61] with bovine serum albumin as a standard. The total number of plants per experimental variant used for the preparation of the necessary mixed samples was 50–60, which provided eight replications for the statistical analysis.

The ascorbate (Asc) content was estimated as described by [62] with some modifications [63]. The total and reduced Asc content was estimated by HPLC using a reverse-phase column (*Watrex Nucleosil 120 5 C18*, *ECOM*, Prague, Czech Republic), 5 mm particle size, 125×4 mm; the solvent system was acetic acid, pH 3, the length of the run was 7 min, the flow rate was 1 mL min^-1^, and the detection wavelength was 244 nm. The percentage of reduced ascorbate (RSA) was then calculated as RSA = 100×reduced Asc/total Asc. Each genotype/water treatment combination was represented by four replications, prepared from mixed samples of ten leaves.

A method using thiols labelled with monobromobimane (mBBr) was used to measure the contents of reduced (GSH) and oxidized (GSSG) glutathione as described by [64]. Bound mBBr was assessed using reverse-phase HPLC (*ECOM*, Prague, Czech Republic) equipped with a fluorescence detector (*Shimadzu RF-10AXL*, *Shimadzu Corporation*, Tokyo, Japan) and separation column *Watrex Nucleosil 120 5 C18*. The standard curve was detected in the range from 0 to 33 nM GSH in 0.1 M HCl. The percentage of reduced glutathione was then calculated as RSG = 100×GSH/(GSH+GSSG). Three replications, prepared from mixed samples of 10 leaves, represented each genotype/water treatment combination.

### Determination of proline content

The content of free proline was determined as described by [65]. Leaves (0.25–0.5 g) were homogenized using a mortar and pestle with 5 mL of 3% sulfosalicylic acid, the homogenate was filtered through filter paper and 1 mL of filtrate was mixed with 1 mL of acid ninhydrin solution and 1 mL of acetic acid. The samples were heated for 30 min, cooled in ice water, followed by the addition of 3 mL of toluene, thorough mixing and a 20-min incubation at room temperature. The upper layer of the separation mixture was used for spectrophotometric measurement of the absorbance at 520 nm (*Anthelie Advanced 2*, *Secomam*, Lyon, France). Ten individual plants were used as independent samples for each genotype/water treatment combination.

### Proteomic analysis

Samples produced from the leaves of 10 plants per genotype/water treatment combination were used for the proteomic analysis. Dried samples containing 100 μg of total protein were dissolved in the sample dissolution buffer. Sample solubilization, reduction, alkylation, trypsin digestion and iTRAQ 8-plex labelling were performed according to the manufacturer's instructions (*Applied Biosystems*, UK). Combined samples were precipitated with 500 μL of acetone overnight at -20°C. The precipitate was spun down, acetone was carefully poured out and the rest of the acetone was left to evaporate for 5 minutes.

The sample was then dissolved in 250 μL of 2 M urea, poured into 17-cm-long focusing tray of *Protean IEF Cell* (*Bio-Rad*, Hercules, CA, USA) and covered with 17-cm IPG strips (pH 3–10, *Bio-Rad*) without paper wicks and oil. Active rehydration at 50 V for 2 hours was followed by voltage steps of 100, 250, 500, 1000 for 15 minutes and a maximum of 10 kV until 40 kVHrs was reached. The current was limited to 50 μA. The strip was cut into pieces 2-3-mm wide. These pieces were sonicated for 15 minutes with 20 μL of 50% acetonitrile (ACN) with 0.1% trifluoroacetic acid (TFA) in parallel. The supernatants were mixed with water (1:1 v/v) and subjected to a nanoreverse-phase HPLC.

LC-MALDI analyses were performed using an *Ultimate 3000* HPLC system (*Dionex*, Sunnyvale, USA) coupled to a *Probot* micro-fraction collector (*Dionex*). Extracted post-IEF fractions were loaded onto a *PepMap 100 C18 RP* column (3 μm particle size, 15 cm long, 75 μm internal diameter; *Dionex*) and separated by a gradient of 5% (v/v) ACN, 0.1% (v/v) TFA to 80% (v/v) ACN, 0.1% (v/v) TFA over a period of 60 min. The flow rate was set to 300 nL/min. The eluate was mixed 1:3 with the matrix solution (2 mg/mL α-cyano-4-hydroxycinnamic acid in 80% ACN) by the *Probot* micro-fraction spotter prior to spotting it onto a MALDI target. The spotting frequency was 5 spots per minute, *i*.*e*., 60 nL eluate + 180 nL matrix solution per MALDI spot.

Spectra were acquired on *4800 Plus MALDI TOF/TOF* analyser (*AB Sciex*, Framingham, USA) equipped with a *Nd*:*YAG* laser (355 nm, firing rate 200 Hz). All spots were first measured in MS mode from m/z 800 to 4,000 and then up to 15 of the strongest precursors were selected for MS/MS analysis which was performed with a collision energy of 1 kV and an operating pressure of the collision cell set to 10^−6^ Torr. Tandem mass spectra were processed with a *4000 Series Explorer* with baseline subtraction enabled (peak width 50), Gaussian smoothing was applied with a filter width of 5, minimum signal to noise of 8, local noise window width of 250 m/z, minimum peak width at full width half max. of 2.9 bins, cluster area signal to noise optimization enabled (threshold 15), and flag monoisotopic peaks enabled (generic formula C_6_H_5_NO).

The database search was performed with *ProteinPilot 4*.*0* (*AB Sciex*) against the database of *Zea mays* protein sequences downloaded from NCBI with trypsin digestion, methyl methanethiosulfonate modification of cysteines, iTRAQ 8-plex labelling, instrument 4800, no special factors, default iTRAQ isotope correction settings, quantitation, bias correction, background correction, biological modifications and thorough ID parameters selected. The probabilities of modifications were not altered. The detected protein threshold (unused protein score and confidence of results) was set to 2.0 and 99.0%, and false discovery rate analysis was enabled. Protein grouping was performed automatically using the default ProGroup^™^ algorithm incorporated in ProteinPilot 4.0. Ratios of iTRAQ for all possible pairs were calculated with default settings. Protein fold change (iTRAQ ratio for an individual protein) was calculated automatically by the software as a weighted average of Log iTRAQ ratios determined for individual peptides belonging to the particular protein after background subtraction. To estimate the false discovery rate (FDR), a decoy database search was performed. The iTRAQ ratios ≥ 2.0 were considered differentially expressed.

The results of the iTRAQ analysis were primarily expressed as several different ratios. The responses of the individual genotypes to stress were evaluated using S_X_/C_X_ ratios where S_X_ represents the drought-stressed plants and C_X_ represents the control plants of the respective genotype; for proteins with decreased levels in stressed plants compared with the control, these ratios were expressed as –1/(S_X_/C_X_). The iTRAQ analysis was also used to compare different behaviours of hybrids and inbred lines, for which contrasts between inbreds and hybrids were expressed by ratios C_F1_/C_P_ and S_F1_/S_P_, where C_F1_, resp. S_F1_, represent the control and stressed plants of the respective F1 hybrid, and C_P_, resp. S_P_, represent the control and stressed plants of the respective parental inbred line. In cases in which a higher protein level was detected in the inbred line, these ratios were expressed as –1/(C_F1_/C_P_), resp. –1/(S_F1_/S_P_) for control and stressed plants, respectively.

### Statistical analysis and evaluation of heterosis

The statistical significance of differences between individual genotype/water treatment combinations was evaluated by one-way analysis of variance followed by Games-Howell tests with a probability level of 0.05 treated as statistically significant. Mid-parent heterosis was calculated as 100×(the mean value of the respective parameter in the F1 hybrid/the average of the mean values of the respective parameter in both parental lines).

## Results

### Differences between control and drought-stressed plants

**Fig 1:**
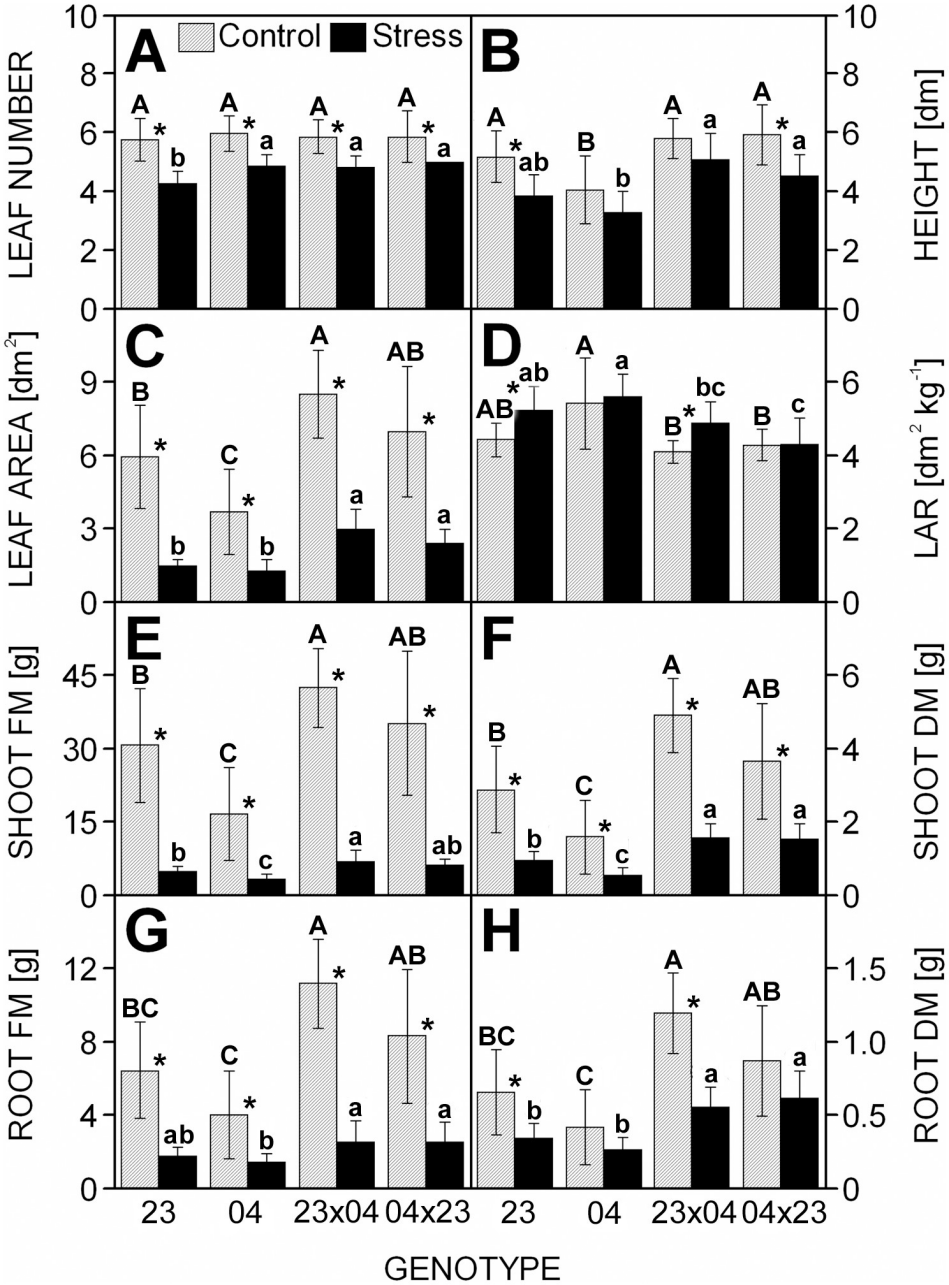
The morphology and biomass characteristics of drought-stressed maize genotypes. The number of fully developed leaves (***A***), the plant height (***B***), the total area of the photosynthetically active leaves (***C***), the leaf area ratio (LAR) (***D***), the shoot fresh mass (FM) (***E***), the shoot dry mass (DM) (***F***), the root fresh mass (***G***) and the root dry mass (***H***) of maize inbred lines 2023 (23) and CE704 (04) and their F1 hybrids 2023×CE704 (23×04) and CE704×2023 (04×23) subjected to 10 days of drought (solid bars) or normally watered (hatched bars). Means ± SD (n = 20) are shown. The letters *A-C* denote the statistical significance of the differences between genotypes under control conditions, the letters *a-c* denote the statistical significance of the differences between genotypes under drought conditions (only those marked with different letters differ significantly at p ≤ 0.05). Asterisks indicate significant differences between control and drought-stressed plants of the respective genotype (p ≤ 0.05).

After 10 days without watering, the drought-stressed plants displayed strong leaf rolling of all leaves with the first two leaves (also the 3^rd^ leaf in the case of the 2023 and 2023×CE704 genotypes) completely dried out and most of the apical parts of their 3^rd^ (in the case of the CE704 and CE704×2023 genotypes) and 4^th^ leaves also dry or yellow. However, the lower leaves of control plants only just started to slightly wilt and their upper leaves (from the 3^rd^ one up) maintained their green colour. All drought-stressed plants were characterized by lower values of various *morphological parameters* such as a plant height, number of fully developed leaves, LA, and shoot and root FM and DM, compared to the control. However, these differences were much less marked or even statistically non-significant in the CE704 inbred line (Fig 1). The F1 hybrid CE704×2023 usually also showed a lower decrease in DM parameters compared to its reciprocal hybrid 2023×CE704 (Fig 1F and 1H). These genotypic differences were reflected in the values of the LAR, which increased due to drought stress in the 2023 and 2023×CE704 genotypes but did not change in the other two genotypes (Fig 1D).

**Fig 2:**
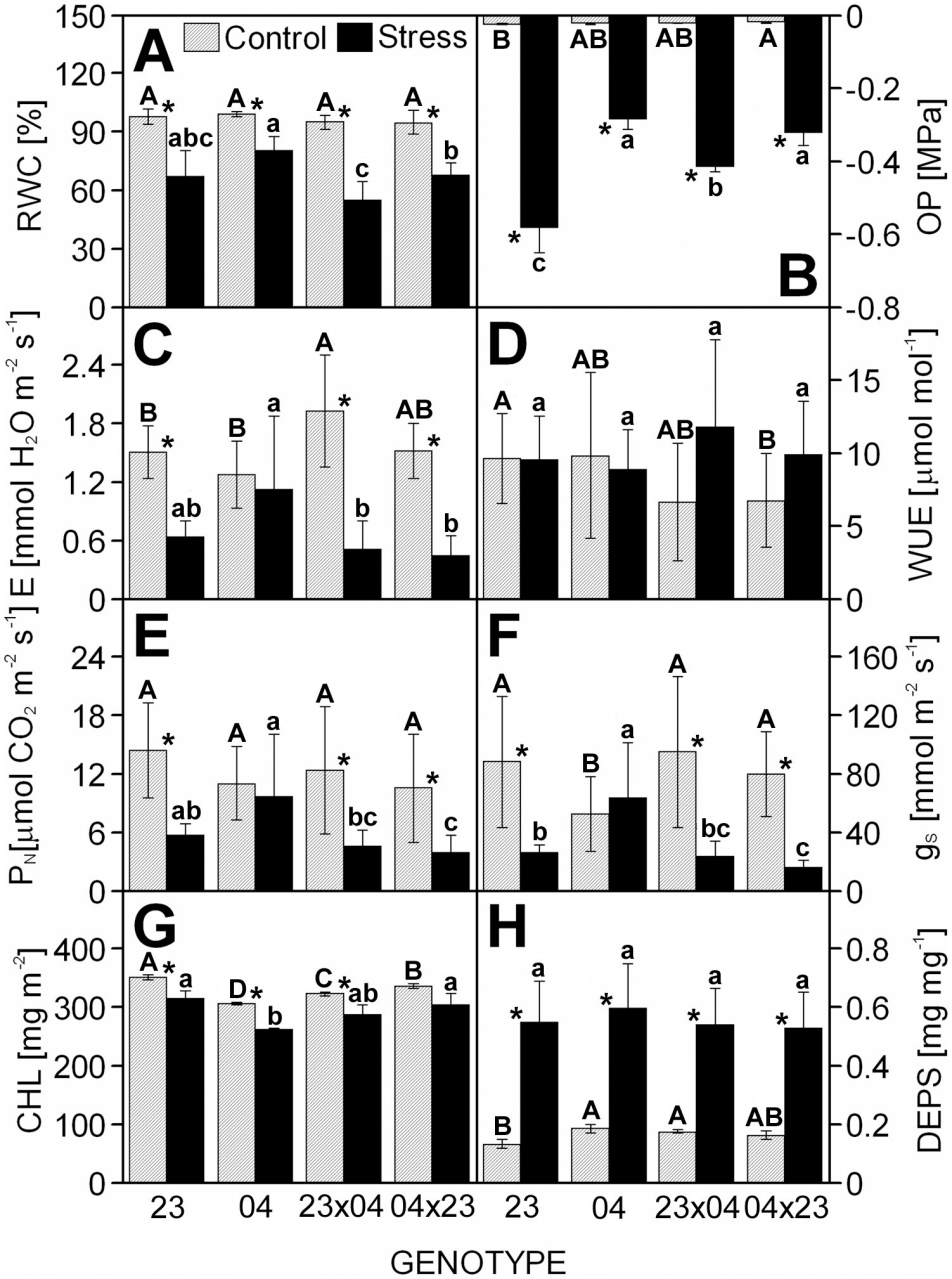
The water use, the gas exchange and the photosynthetic pigment characteristics in leaves of drought-stressed maize genotypes. The relative water content (RWC) (***A***), the leaf osmotic potential (OP) (***B***), the net transpiration rate (E) (***C***), the water use efficiency (WUE) (***D***), the net photosynthetic rate (P_N_) (***E***), the stomatal conductance (g_S_) (***F***), the total chlorophyll (Chl) content (***G***) and the deepoxidation state of xanthophyll cycle pigments (DEPS) (***H***) in leaves of maize inbred lines 2023 (23) and CE704 (04) and their F1 hybrids 2023×CE704 (23×04) and CE704×2023 (04×23) subjected to 10 days of drought (solid bars) or normally watered (hatched bars). Means ± SD (n = 10 for RWC, 5 for OP, 22–28 for gas exchange characteristics and 6 for photosynthetic pigments´ characteristics) are shown. The letters *A-D* denote the statistical significance of the differences between genotypes under control conditions, the letters *a-c* denote the statistical significance of the differences between genotypes under drought conditions (only those marked with different letters differ significantly at p ≤ 0.05). Asterisks indicate significant differences between control and drought-stressed plants of the respective genotype (p ≤ 0.05).

The different response of the CE704 inbred line to drought was evident not only for the morphological parameters but also for parameters associated with *plant water management*. The RWC and the OP decreased due to drought, but this decrease was lower in the CE704 genotype (Fig 2A and 2B). The E also decreased in all examined genotypes with the exception of CE704 (Fig 2C) and the same phenomenon was observed for the g_S_ and the P_N_ (Fig 2E and 2F). A more detailed analysis of the gas exchange parameters made in days 0, 2, 4, 6 and 8 after the beginning of drought period showed that the 2023 inbred line is characterized by the earliest reduction of g_S_, E and P_N_ with the 2023×CE704 hybrid closely following (S2 File). The drought-exposed plants of the CE704 inbred line maintained these parameters at a level higher or at least comparable with the control during the whole course of the experiment and the behaviour of the CE704×2023 hybrid was to some extent similar to its maternal parent (S2 File). However, the WUE did not significantly change in either genotype (Fig 2D). The reciprocal F1 hybrids significantly differed in terms of drought-induced change in RWC and OP with reduced change observed in CE704×2023 (Fig 2A and 2B).

**Fig 3:**
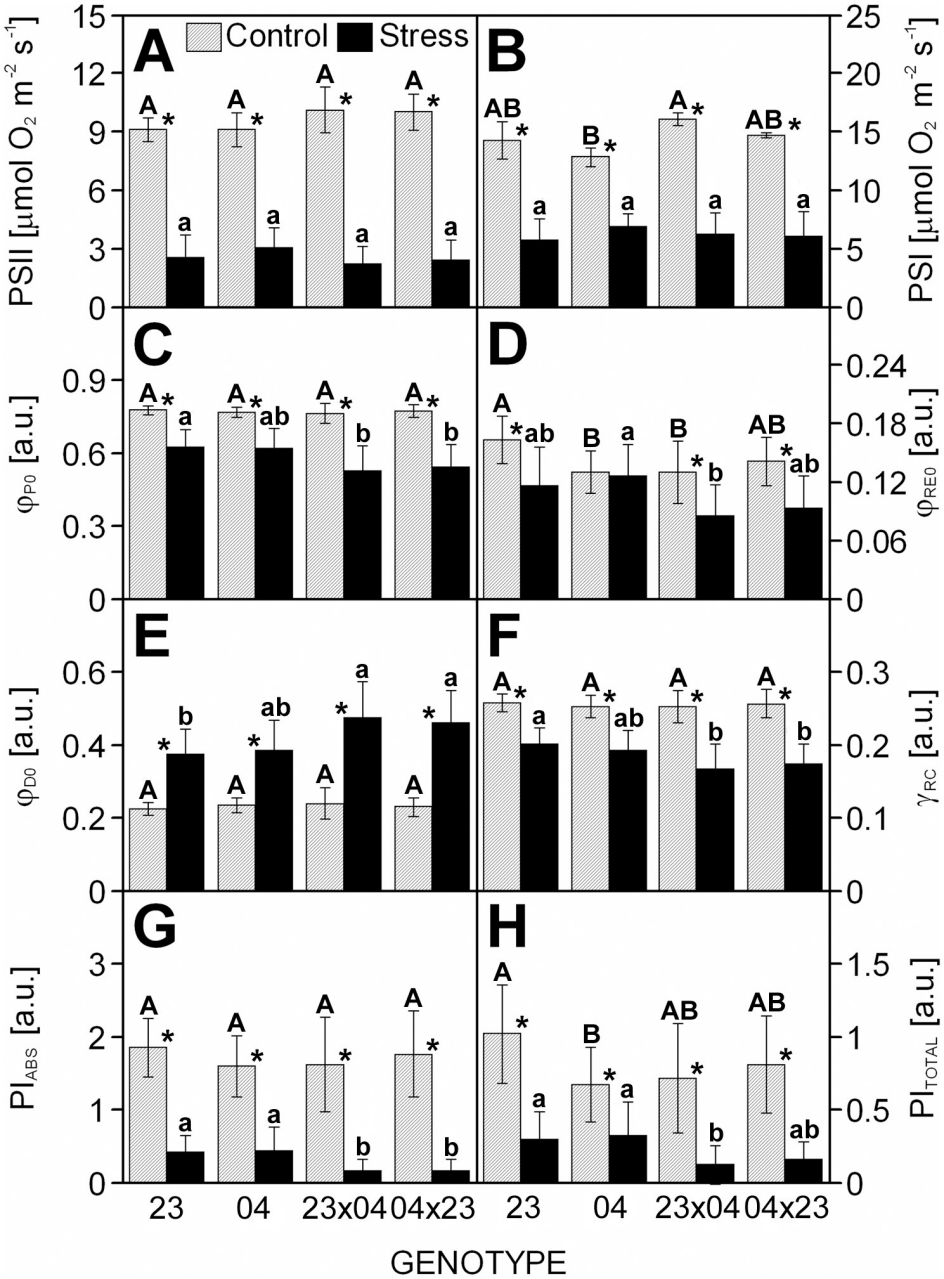
The characteristics of primary photosynthetic processes in leaves of drought-stressed maize genotypes. The activities of Photosystem (PS) II (***A***) and PSI (***B***) in isolated mesophyll chloroplasts, the maximum quantum yield of primary PSII photochemistry (φ_P0_) (***C***), the quantum yield of electron transport flux until the PSI electron acceptors (φ_RE0_) (***D***), the quantum yield of energy dissipation (φ_D0_) (***E***), the probability that a PSII chlorophyll functions as reaction center (γRC) (***F***), the performance index for energy conservation from photons absorbed by PSII antenna, to the reduction of Q_B_ (PI_ABS_) (***G***) and the performance index for energy conservation from photons absorbed by PSII antenna, until the reduction of PSI acceptors (PI_TOTAL_) (***H***) in leaves of maize inbred lines 2023 (23) and CE704 (04) and their F1 hybrids 2023×CE704 (23×04) and CE704×2023 (04×23) subjected to 10 days of drought (solid bars) or normally watered (hatched bars). Means ± SD (n = 4 for PSII and PSI activities in isolated chloroplasts, and 20 for chlorophyll fluorescence parameters) are shown. The letters *A-B* denote the statistical significance of the differences between genotypes under control conditions, the letters *a-b* denote the statistical significance of the differences between genotypes under drought conditions (only those marked with different letters differ significantly at p ≤ 0.05). Asterisks indicate significant differences between control and drought-stressed plants of the respective genotype (p ≤ 0.05).

**Fig 4:**
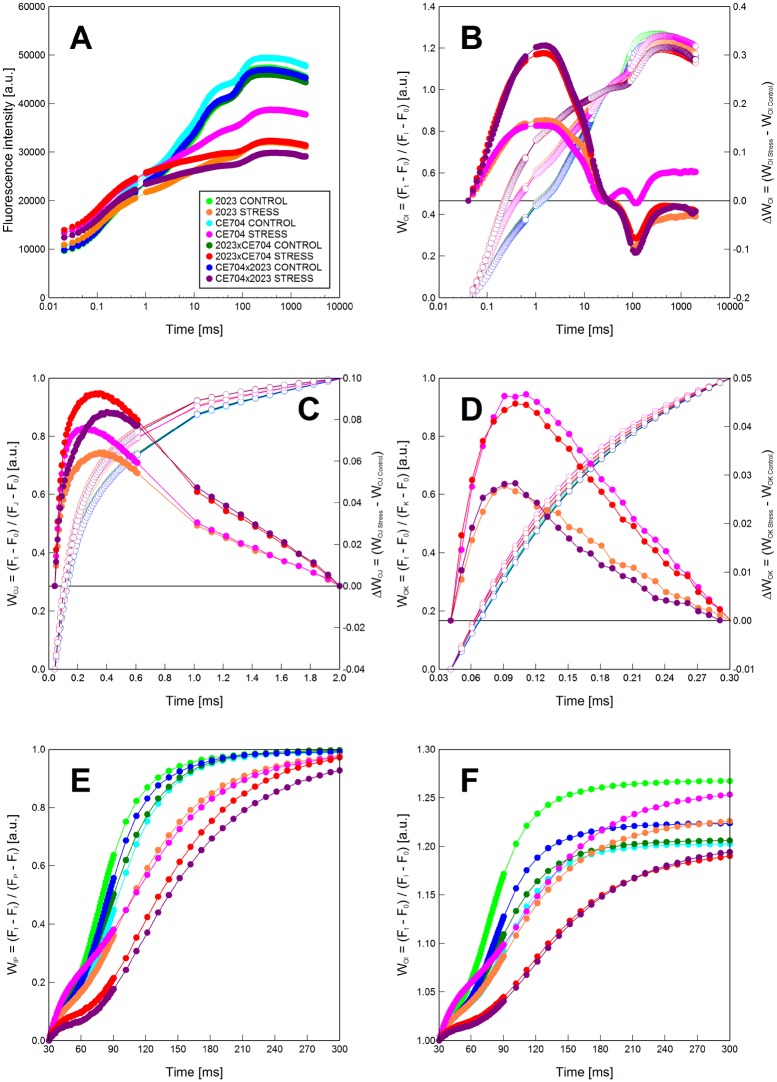
The chlorophyll *a* fluorescence kinetics (O-J-I-P) measured in dark-adapted leaves of drought-stressed maize genotypes. Direct transients (***A***), the relative variable fluorescence and the difference kinetics W_OI_ = (F_t_-F_0_)/(F_I_-F_0_) and ΔW_OI_ = (W_OI Stress_-W_OI Control_) (***B***), W_OJ_ = (F_t_-F_0_)/(F_J_-F_0_) and ΔW_OJ_ = (W_OJ Stress_-W_OJ Control_) (***C***), W_OK_ = (F_t_-F_0_)/(F_K_-F_0_) and ΔW_OK_ = (W_OK Stress_-W_OK Control_) (***D***), W_IP_ = (F_t_-F_I_)/(F_P_-F_I_) (***E***) and the part of W_OI_ between 30 and 300 ms (***F***) in leaves of maize inbred lines 2023 and CE704 and their F1 hybrids 2023×CE704 and CE704×2023 subjected to 10 days of drought (Stress) or normally watered (Control). The relative variable fluorescence is plotted on left vertical axes using open symbols, the difference kinetics is plotted on right vertical axes using solid symbols. F_t_ represents the fluorescence intensity measured at any time during the recording period, F_I_ the fluorescence intensity at the I-step, F_J_ the fluorescence intensity at the J-step, F_K_ the fluorescence intensity at the K-step, F_P_ the maximum fluorescence intensity, and F_0_ the initial fluorescence intensity. Mean values (n = 20) are shown for each genotype/water treatment combination.

The content of Chls significantly decreased in all drought-stressed plants with the exception of CE704×2023, while DEPS markedly increased due to drought (Fig 2G and 2H). The same result was found for most parameters associated with *primary photosynthetic processes* (Fig 3). Drought caused a significant decrease in the photochemical activities/quantum yields/performance indices characterizing either PSII (Fig 3A, 3C and 3G), PSI (Fig 3B) or the whole electron-transport chain (Fig 3D and 3H). Only the CE704 inbred line maintained the same quantum yield of the whole electron-transport chain under drought and control conditions (Fig 3D). Drought also caused a decrease in the probability that a PSII Chl molecule functions as a reaction centre Chl (Fig 3F) and an increase in the dissipation of the excess excitation energy (Fig 3E). However, the drought-tolerant CE704 inbred line again showed a better acclimation to drought demonstrated by its smallest change in Chl fluorescence intensity as seen in the O-J-I-P transients (Fig 4A). Drought stress resulted in a positive K-band in all four examined genotypes, which was visible in the plot of difference kinetics ΔW_OJ_ (Fig 4C). This indicated either an inactivation of the oxygen-evolving complex (OEC) and/or an increase in the size of a functional PSII antenna. The L-band revealed by difference kinetics ΔW_OK_ was also in the positive range for all drought-stressed genotypes, which reflected a lower excitonic connectivity (functional grouping) of the individual PSII units. In this case, the drought-sensitive 2023 inbred line was characterized by better connectivity under stress conditions compared with the drought-resistant CE704 (Fig 4D). Regarding the rate of reduction of the end electron acceptors at the PSI acceptor side, a decrease was observed in drought-stressed plants of all genotypes (as seen from the shift of W_IP_ curves to the right). There were no marked differences between both parental lines whereas F1 hybrids were more affected (Fig 4E). The size of the pool of these electron acceptors decreased due to drought in inbred line 2023 (which also had the largest pool of these acceptors in control conditions among all examined genotypes) but not in the CE704 genotype. This result was demonstrated by the lower position of the curve of normalized fluorescence data W_OI_ plotted in the 30–300 ms time range in 2023 (Fig 4F). Both F1 hybrids responded to drought similarly and did not differ in any of these parameters except for the L-band amplitude, for which the 2023×CE704 hybrid was characterized by a higher amplitude, *i*.*e*. a lower excitonic connectivity between PSII units, compared to its reciprocal hybrid CE704×2023 (Fig 4).

**Fig 5:**
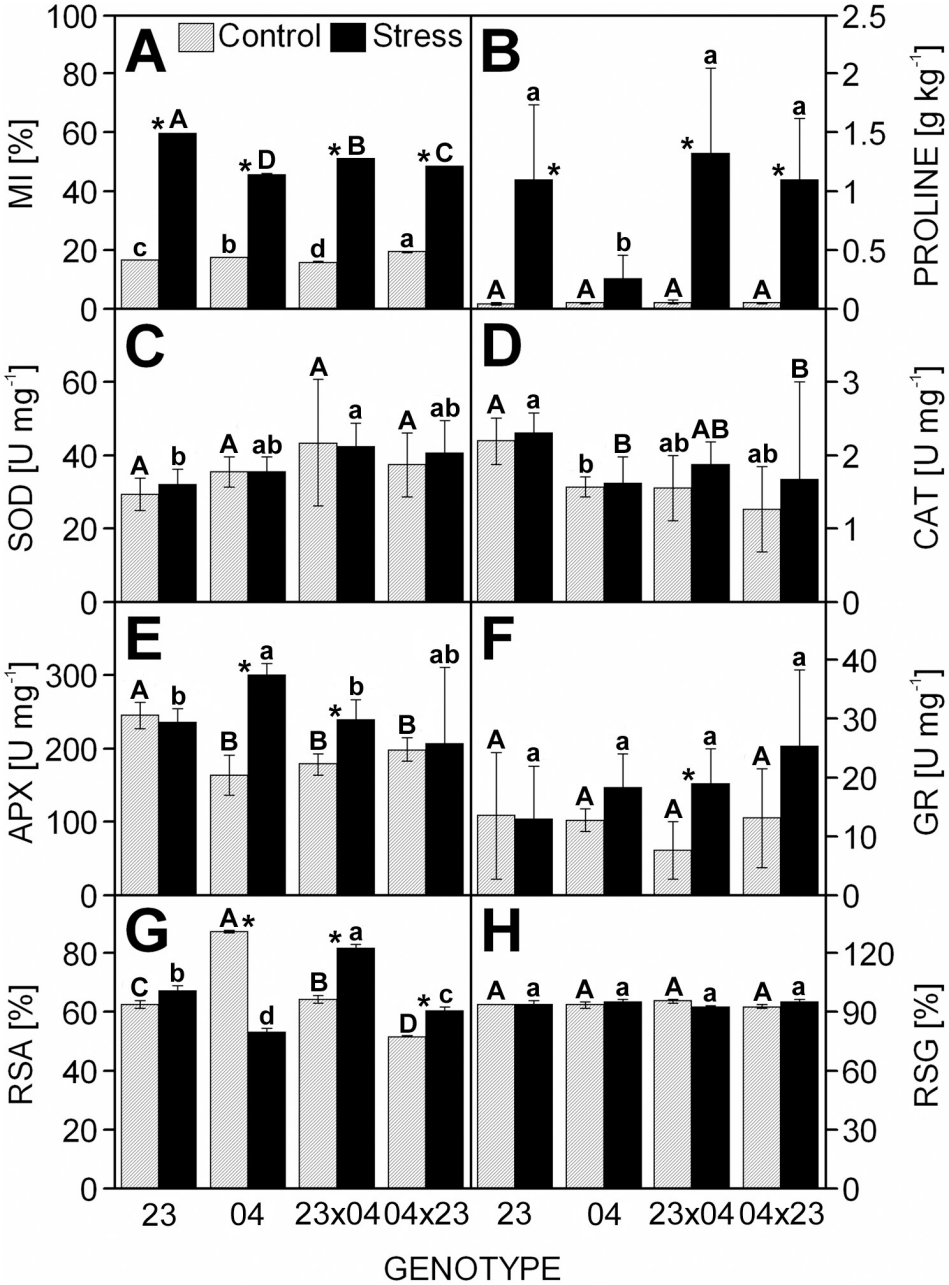
The cell membrane injury, the activities/contents of antioxidants and the proline content in leaves of drought-stressed maize genotypes. The cell membrane injury (MI) (***A***), the content of proline (***B***), the activities of superoxide dismutase (SOD) (***C***), catalase (CAT) (***D***), ascorbate peroxidase (APX) (***E***), glutathione reductase (GR) (***F***), the percentage of reduced ascorbate (RSA) (***G***) and the percentage of reduced glutathione (RSG) (***H***) in leaves of maize inbred lines 2023 (23) and CE704 (04) and their F1 hybrids 2023×CE704 (23×04) and CE704×2023 (04×23) subjected to 10 days of drought (solid bars) or normally watered (hatched bars). Means ± SD (n = 5 for MI, 10 for the proline content, 8 for the activities of antioxidant enzymes, 4 for RSA and 3 for RSG) are shown. The letters *A-D* denote the statistical significance of the differences between genotypes under control conditions, the letters *a-d* denote the statistical significance of the differences between genotypes under drought conditions (only those marked with different letters differ significantly at p ≤ 0.05). Asterisks indicate significant differences between control and drought-stressed plants of the respective genotype (p ≤ 0.05).

Regarding the *plant protection parameters*, the CE704 inbred line was characterized by a slightly lower increase in MI after exposure to drought stress conditions in comparison to the other three genotypes. The change in proline content due to drought was actually statistically non-significant for this inbred line (Fig 5A and 5B). The inbred line CE704 also showed a marked and statistically significant increase in APX activity (Fig 5E) and a significant decrease in RSA due to drought (Fig 5G) whereas the drought-stressed plants of both F1 hybrids displayed significantly higher RSA values compared with the control ones (Fig 5G). The activities of SOD, CAT or GR did not show any significant drought-induced change in either of the examined genotypes (with the exception of the GR activity in the 2023×CE704 hybrid), which was also observed for RSG (Fig 5C, 5D, 5F and 5H). The differences between the reciprocal F1 hybrids were almost absent for most plant protection parameters examined with the exception of RSA (Fig 5). The MI values showed a slightly lower increase in CE704×2023 compared with 2023×CE704 (Fig 5A).

The iTRAQ analysis of *leaf proteome* identified 857 proteins that were matched in the NCBI database, including 297 proteins with at least a two fold change in response to drought stress in at least one genotype. These proteins were classified into 13 groups based on their functions (S1 Table). In addition to proteins with various or unknown functions (“Miscellaneous” category, 21.2%), the most-represented groups were proteins associated with primary photosynthetic processes (17.2%), proteins involved in saccharide metabolism (15.8%) and proteins participating in gene expression and its regulation (14.1%).

**Fig 6:**
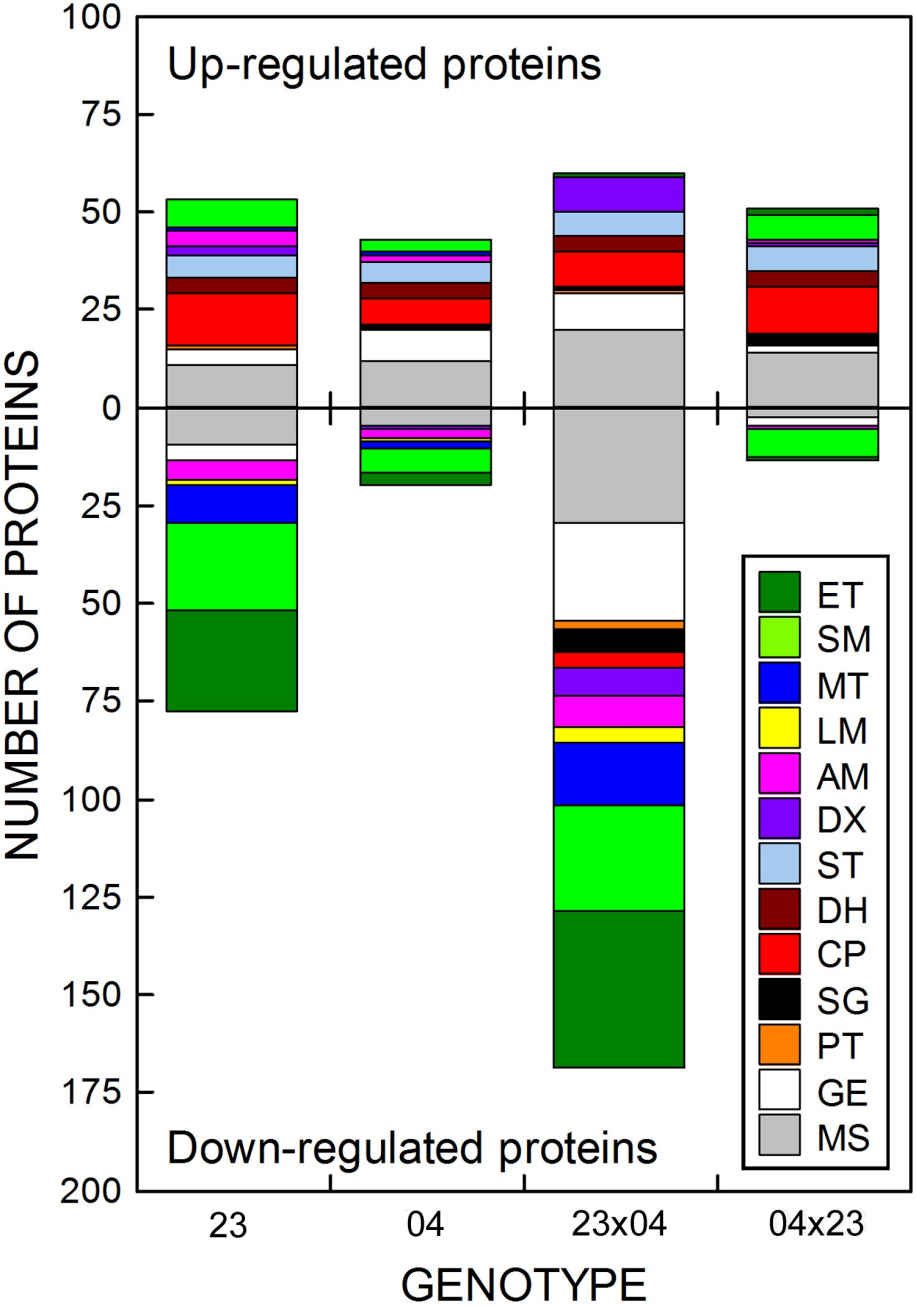
The functional classification of differentially expressed drought-related proteins from maize leaves. The number of proteins with up-regulated or down-regulated levels due to drought stress identified by the iTRAQ method in leaves of maize inbred lines 2023 (23) and CE704 (04) and their F1 hybrids 2023×CE704 (23×04) and CE704×2023 (04×23) is shown; only those proteins whose levels changed due to drought by at least two fold were included. ET: proteins of the photosynthetic light-harvesting, electron-transport chain and chlorophyll synthesis; SM: proteins participating in photosynthetic carbon fixation and saccharide metabolism; MT: membrane proteins participating in transport and energetics; LM: proteins participating in lipid metabolism; AM: proteins participating in amino acid metabolism; DX: detoxification proteins; ST: stress proteins; DH: dehydrins; CP: chaperones; SG: proteins involved in cell signalling; PT: proteases and their inhibitors; GE: proteins participating in gene expression and its regulation; MS: miscellaneous proteins.

The *numbers of proteins* that were up-regulated by drought were quite similar in all genotypes (43–60 proteins with at least a two fold change in their levels associated with drought stress). These proteins were represented mostly by chaperones, dehydrins, stress proteins or (particularly in CE704 and 2023×CE704 genotypes) proteins involved in the regulation of gene expression (Fig 6). More differences among genotypes in their response to drought stress were observed for the down-regulated proteins. The inbred line CE704 and its CE704×2023 hybrid were characterized by only a few (20, resp. 14) down-regulated proteins. In contrast, the other two genotypes showed strong down-regulation of the protein level. This phenomenon applied particularly to the 2023×CE704 hybrid, in which almost 170 proteins changed their level of expression to at least two fold due to drought stress (Fig 6). Interestingly, only 45 proteins were shared between the two groups of 169 and 78 proteins that were down-regulated in the 2023×CE704 and the 2023 genotypes, respectively (S1 Table). Most down-regulated proteins belonged to the categories of photosynthetic electron-transport chain components (and associated proteins) or proteins that participate in photosynthetic carbon fixation and saccharide metabolism. Genotypes 2023 and 2023×CE704 also showed a decrease in the levels of a rather large number of membrane proteins involved in transport and proteins that participate in amino acid metabolism, and the 2023×CE704 additionally also in proteins belonging to the “Detoxification”, “Cell signalling” and “Gene expression and its regulation” (particularly ribosomal proteins) groups (Fig 6, S1 Table).

Almost all proteins that showed the highest accumulation during dehydration belonged to the categories of “Dehydrins” or “Chaperones”, as shown in Table 1 which shows *the most extreme responses* to drought stress, *i*.*e*., five proteins with levels that were the most strongly up-regulated in individual genotypes. In contrast, the most strongly down-regulated proteins differed between the genotypes (Table 2). Some of these proteins were involved in CO_2_ fixation (this applied particularly to the 2023 inbred line) and photosynthetic electron-transport chain, others had various functions. Tables 1 and 2 also show the genotypic differences in the actual *levels* of up- or down-regulation of the differentially-expressed proteins. The highest up-regulation was observed in the drought-sensitive inbred line 2023 (up to almost 32-fold for some dehydrins and RAB-17 protein) whereas the drought-resistant line CE704 showed the smallest increase in protein levels amongst all examined genotypes (Table 1, S1 Table). On the other hand, the highest level of down-regulation was observed in the hybrid 2023×CE704, in which the chloroplastic ribosomal protein S4 displayed almost a 43-fold decrease due to drought and two subunits of PSII (PsbA and PsbE) showed 25- to 30-fold decrease compared to the control plants. The 2023 inbred line was characterized by a strong down-regulation of proteins associated with photosynthetic carbon fixation (pyruvate, *ortho*phosphate dikinase (PPDK), glyceraldehyde-3-phosphate dehydrogenase (GAPDH) and CP12 protein of Calvin cycle) and the PsbH subunit of the PSII complex (16-18-fold). Both CE704 and CE704×2023 genotypes displayed only a slight decrease of protein levels (no more than 4-fold) (Table 2, S1 Table).

**Table 1:** Five most strongly up-regulated proteins in drought-stressed maize plants of two inbred lines (2023, CE704) and their F1 hybrids (2023×CE704, CE704×2023).

Protein	Matching sequence (EST/protein)	23	04	23×04	04×23
**Ranked according to the 2023 genotype**					
Dehydrin 13	gi|195625830	**31.9**	9.0	20.7	9.5
Dehydrin RAB-17	gi|223950115	**29.4**	11.2	18.9	12.7
Dehydrin	gi|532623	**24.4**	9.0	11.7	7.2
Late embryogenesis abundant protein (group 3)	gi|7387829	**17.1**	6.4	14.1	17.1
Hypothetical protein	gi|195655323	**15.0**	1.6	4.1	4.1
**Ranked according to the CE704 genotype**					
Dehydrin RAB-17	gi|223950115	29.4	**11.2**	18.9	12.7
Dehydrin COR410	gi|226532838	11.3	**9.5**	13.7	4.7
Dehydrin	gi|532623	24.4	**9.0**	11.7	7.2
Dehydrin 13	gi|195625830	31.9	**9.0**	20.7	9.5
Protein binding protein	gi|238011090	1.3	**6.8**	-1.6	2.8
**Ranked according to the 2023×CE704 genotype**					
Dehydrin 13	gi|195625830	31.9	9.0	**20.7**	9.5
Dehydrin RAB-17	gi|223950115	29.4	11.2	**18.9**	12.7
Late embryogenesis abundant protein (group 3)	gi|7387829	17.1	6.4	**14.1**	17.1
Dehydrin COR410	gi|226532838	11.3	9.5	**13.7**	4.7
Heat shock 70 kDa protein 4-like	gi|308081377 ^BD^	8.2	5.8	**13.2**	6.2
**Ranked according to the CE704x2023 genotype**					
Late embryogenesis abundant protein (group 3)	gi|7387829	17.1	6.4	14.1	**17.1**
Dehydrin RAB-17	gi|223950115	29.4	11.2	18.9	**12.7**
Heat shock protein (17.4 kDa, class I)	gi|296512733	10.2	4.5	13.1	**11.4**
Dehydrin 13	gi|195625830	31.9	9.0	20.7	**9.5**
Dehydrin	gi|532623	24.4	9.0	11.7	**7.2**

The numbers in the individual columns represent the n-fold increase in the protein content after 10 days of drought, as derived from the iTRAQ analysis ratios (negative values represent the n-fold decrease). 23 represents the 2023, 04 represents the CE704, 23×04 represents the 2023×CE704 and 04×23 represents the CE704×2023. BD: *Brachypodium distachyon* L.

**Table 2:** Five most strongly down-regulated proteins in drought-stressed maize plants of two inbred lines (2023, CE704) and their F1 hybrids (2023×CE704, CE704×2023).

Protein	Matching sequence (EST/protein)	23	04	23×04	04×23
**Ranked according to the 2023 genotype**					
Glyceraldehyde-3-phosphate dehydrogenase B	gi|238011684	**-18.3**	-1.6	-5.8	-2.9
Calvin cycle protein CP12-1	gi|226493683	**-18.1**	-2.4	-1.3	-3.6
PSII subunit—PsbH	gi|902250	**-16.6**	-2.6	-4.7	-3.2
Pyruvate,*ortho*phosphate dikinase	gi|257659143	**-16.0**	-2.3	-4.8	-2.2
ATP synthase β subunit (chloroplastic)	gi|902229	**-9.6**	-1.5	-8.0	-1.5
**Ranked according to the CE704 genotype**					
Phosphoenolpyruvate carboxykinase [ATP]	gi|291048562	-4.2	**-3.3**	-3.8	-3.4
UDP-sulfoquinovose synthase	gi|238014584	-1.8	**-2.8**	-2.2	1.1
Catalase 3 isoform 1	gi|257675731	2.2	**-2.7**	1.6	1.5
Rubisco activase small isoform	gi|313574198	-3.8	**-2.6**	-3.1	-2.1
Cytochrome *b*_*6*_*f* complex subunit (cytochrome b_*6*_)	gi|902251	-2.3	**-2.6**	-4.5	-1.5
**Ranked according to the 2023×CE704 genotype**					
Ribosomal protein S4 (chloroplastic)	gi|902224	-1.1	1.3	**-42.9**	-1.3
PSII subunit—PsbE (cytochrome *b*_*559*_ α)	gi|902238	-2.3	-1.7	**-29.9**	-1.4
PSII subunit—PsbA (D1 protein)	gi|902201	-2.0	-1.2	**-25.8**	-1.5
Triose phosphate/phosphate translocator precursor (chloroplastic)	gi|126633328	-1.2	1.9	**-12.8**	1.2
ATP-dependent Clp protease proteolytic subunit	gi|226529931	-1.7	-1.5	**-12.7**	-1.4
**Ranked according to the CE704x2023 genotype**					
Calvin cycle protein CP12-1	gi|226493683	-18.2	-2.4	-1.3	**-3.6**
Phospho*enol*pyruvate carboxykinase [ATP]	gi|291048562	-4.2	-3.3	-3.8	**-3.4**
PSII subunit—PsbH	gi|902250	-16.6	-2.6	-4.7	**-3.2**
Thiamine biosynthetic enzyme	gi|596080	-4.7	-2.1	-10.1	**-3.1**
Glyceraldehyde-3-phosphate dehydrogenase B	gi|238011684	-18.3	-1.6	-5.8	**-2.9**

The numbers in the individual columns represent the n-fold decrease in the protein content after 10 days of drought, as derived from the iTRAQ analysis ratios (positive values represent n-fold increase). 23 represents the 2023, 04 represents the CE704, 23×04 represents the 2023×CE704 and 04×23 represents the CE704×2023.

### Differences between F1 hybrids and their inbred parental lines

Both F1 hybrids showed strong positive heterosis (often between 150–200% of the mid-parent value) for various *morphological parameters* under control conditions. The 2023×CE704 hybrid was usually characterized by higher values of the heterotic effect compared to the CE704×2023 (Table 3). In some cases, heterosis increased in drought-stressed plants; however, this did not always apply to both F1 hybrids. Mid-parent heterosis for root DM and FM and shoot DM decreased after drought stress in the 2023×CE704 hybrid, but it did not change or even markedly increased in the CE704×2023 hybrid. The reverse situation was found for plant height. However, positive heterosis for the shoot FM, which was observed in both F1 hybrids under control conditions, only marginally changed when the plants were exposed to 10 days of drought simulation, and heterosis in the LA increased after exposure to drought similarly in both F1 hybrids (Table 3).

**Table 3:** Mid-parent heterosis in selected morphological, physiological and biochemical parameters of drought-stressed maize.

Parameter	Control		Stress	
	23×04	04×23	23×04	04×23
Number of fully developed leaves	100.00	100.00	105.49	109.89
Plant height	126.07	128.63	143.08	127.35
Leaf area	177.10	144.24	216.47	174.48
Leaf area ratio	82.86	86.77	90.32	79.49
Fresh mass of shoot	180.30	149.38	170.19	150.58
Dry mass of shoot	219.71	163.25	209.59	206.76
Fresh mass of roots	215.01	159.63	155.36	157.66
Dry mass of roots	223.62	161.81	181.98	202.90
Leaf relative water content	96.51	96.29	74.56	92.14
Leaf osmotic potential	91.36	83.13	95.67	74.51
Transpiration rate	138.77	109.01	57.65	50.23
Water use efficiency	68.06	69.22	128.45	107.64
Stomatal conductance	134.95	113.40	53.16	36.13
Net photosynthetic rate	97.30	82.92	59.57	51.81
PSI activity	118.75	108.22	98.59	95.32
PSII activity	111.12	109.97	80.03	85.66
Maximum quantum yield of primary PSII photochemistry	98.76	99.97	85.09	87.19
Quantum yield for reduction of end electron acceptors at the PSI side	88.59	96.21	70.31	77.00
Quantum yield of energy dissipation	104.21	100.07	124.38	120.92
Probability that a PSII chlorophyll molecule functions as a reaction center	99.16	100.62	85.08	88.42
PI_ABS_	94.04	101.85	36.72	39.11
PI_TOTAL_	84.47	95.66	38.97	50.67
Chlorophyll (*a*+*b*) content	98.25	102.17	99.91	105.91
Deepoxidation state of xanthophyll cycle pigments	110.32	102.32	94.43	92.38
Membrane injury index	92.72	113.94	96.53	92.02
Free proline content	126.82	108.36	196.52	162.75
Superoxide dismutase activity	134.48	115.75	125.52	120.25
Catalase activity	82.04	67.09	95.17	85.18
Ascorbate peroxidase activity	87.30	97.16	88.84	76.87
Glutathione reductase activity	58.25	100.34	121.30	162.67
Reduced/total ascorbate ratio	85.67	68.75	136.58	100.56
Reduced/total glutathione ratio	101.86	99.09	97.74	100.59

Two reciprocal F1 hybrids (23×04: 2023×CE704, 04×23: CE704×2023) were evaluated under conditions of normal water supply (Control) or after 10 days of drought (Stress). PI_ABS_, resp. PI_TOTAL_: performance indices for energy conservation from exciton to the reduction of the Q_B_ plastoquinone, resp. the end electron acceptors of the thylakoid electron-transport chain. PS: Photosystem.

Regarding the parameters associated with *plant water management*, positive mid-parent heterosis was observed only exceptionally, *e*.*g*. for the E and the g_S_ under control conditions and for the WUE under drought conditions (Table 3). In all these cases, the 2023×CE704 hybrid was characterized by higher heterosis (approx. 130%) than its reciprocal genotype CE704×2023 (approx. 110%). Mid-parent heterosis usually strongly decreased (often to approx. half the values of the parental mean) after exposure of plants to drought, the same was observed for the P_N_ (Table 3). This phenomenon was caused by the different behaviour of the CE704 inbred line in comparison to the other genotypes as described above (Fig 2).

Strong negative (36–50% of the parental mean) mid-parent heterosis was observed for both performance indices derived from the Chl *a* fluorescence measurements and characterizing *primary photosynthetic processes* in the drought-stressed plants. Regarding other Chl *a* fluorescence parameters or photosynthetic pigment contents or ratios, the F1 hybrids usually did not differ greatly from their parents under control conditions and showed none or slightly negative mid-parent heterosis under stress conditions (Table 3). However, the stressed plants of both F1 hybrids showed positive mid-parent heterosis for the quantum yield of energy dissipation and the values of this parameter in both hybrids were significantly higher in comparison to the 2023 inbred line under these conditions (Table 3, Fig 3E). The F1 hybrids were also characterized by a slightly increased inactivation of OEC as observed by the amplitude of the K-band (Fig 4C), a lower rate of reduction of the PSI end electron acceptors and a smaller pool of these acceptors compared with their parents (Fig 4E and 4F). The 2023×CE704 hybrid showed a similar drought-caused decrease in the excitonic connectivity between individual PSII units as its paternal parent CE704, whereas the reciprocal hybrid CE704×2023 imitated its paternal parent 2023 in this respect (Fig 4D). Both F1 hybrids were also characterized by slightly positive (approx. 110–120%) mid-parent heterosis for the PSI and PSII activities measured in chloroplasts isolated from the control plants; however, this changed under drought stress conditions, in which either none or even negative heterosis was observed. In any case, the hybrid-inbred differences in these activities were statistically non-significant (Table 3, Fig 3A and 3B).

Both F1 hybrids displayed positive mid-parent heterosis in the content of free proline under control conditions; this heterosis markedly increased (to approx. 160–200% of the parental mean) after 10 days of drought simulation (Table 3). Again, this was caused by the different behaviour of the CE704 genotype (Fig 5B). Positive heterosis was also observed for SOD activity, but it did not display a large change due to drought and the differences between hybrids and their inbred parental lines were mostly statistically non-significant (Table 3, Fig 5C). Regarding the other parameters associated with *plant protection* against stress, mid-parent heterosis for the activities of other antioxidant enzymes was mostly either negative or none and the same applied for the RSA and the RSG (Table 3). The drought-stressed plants of both F1 hybrids (particularly the CE704×2023 genotype) showed positive mid-parent heterosis for the activity of GR, but the hybrid-inbred differences were statistically non-significant (Table 3, Fig 5F). The 2023×CE704 hybrid (but not the CE704×2023) was also characterized by positive heterosis for the RSA parameter when grown under stress conditions, which was due to the different behaviour of the CE704 inbred line compared to the other examined genotypes (Table 3, Fig 5G).

Amongst 857 proteins identified by iTRAQ analysis of *leaf proteome* and matched in the NCBI database, 268 proteins showed at least a two fold difference between at least one hybrid and one of its parents in either control or stress conditions (or both). The functional classification of these proteins revealed that the most highly represented proteins belonged to the “Miscellaneous” category (21.3%) together with proteins associated with primary photosynthetic processes (17.9%), proteins involved in saccharide metabolism (15.3%) and proteins participating in gene expression and its regulation (12.7%) (S1 Table).

**Fig 7:**
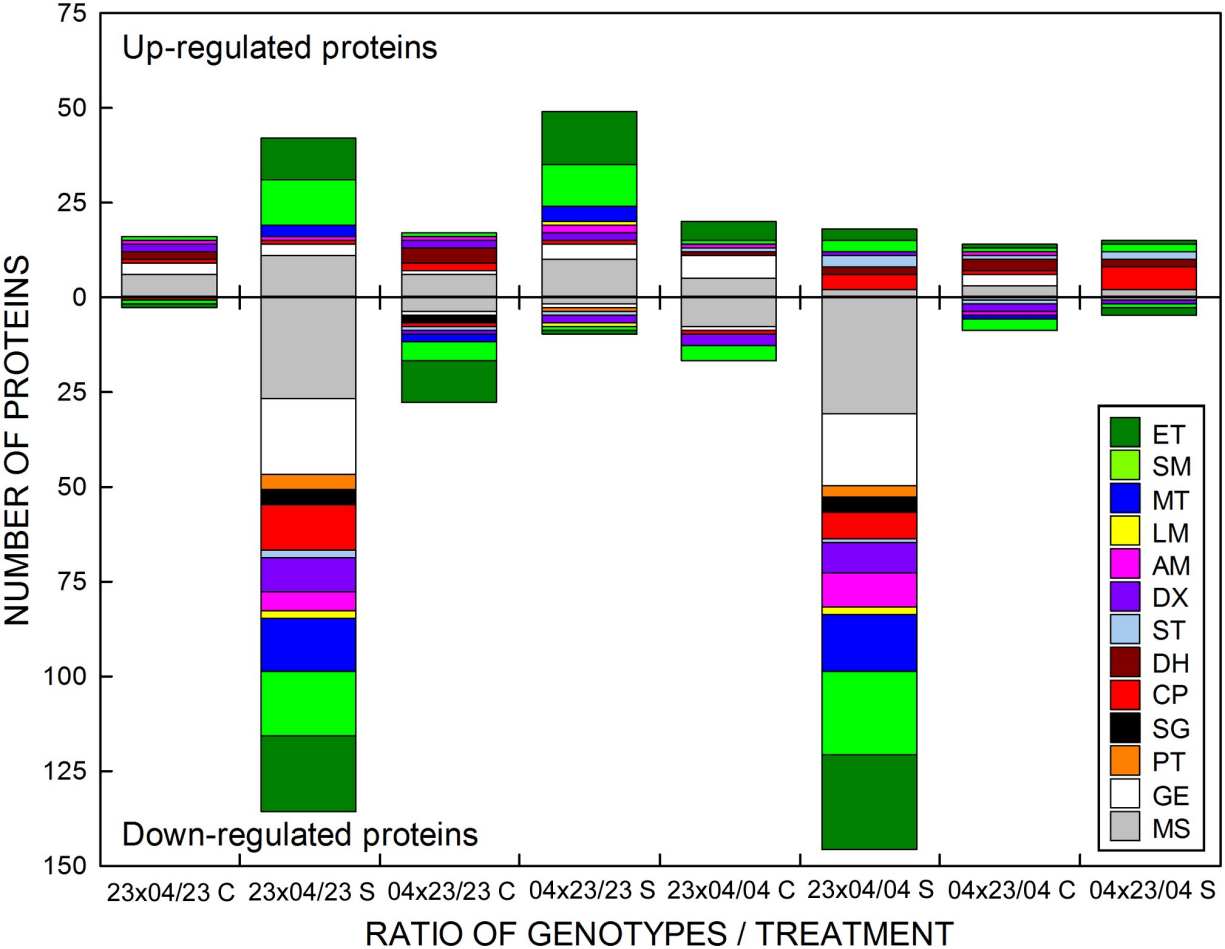
The functional classification of proteins from maize leaves with different responses in parental lines and F1 hybrids. The number of proteins with higher or lower level in F1 hybrids (as compared to parental lines) identified by an iTRAQ method in leaves of normally watered (C) or drought-stressed (S) plants of maize is shown; only those proteins whose levels differred between the respective hybrid and its parental line by at least two fold were included. 23: drought-sensitive parental inbred line 2023, 04: drought-tolerant parental inbred line CE704, 23×04: 2023×CE704 hybrid, 04×23: CE704×2023 hybrid. ET: proteins of the photosynthetic light-harvesting, electron-transport chain and chlorophyll synthesis; SM: proteins participating in photosynthetic carbon fixation and saccharide metabolism; MT: membrane proteins participating in transport and energetics; LM: proteins participating in lipid metabolism; AM: proteins participating in amino acid metabolism; DX: detoxification proteins; ST: stress proteins; DH: dehydrins; CP: chaperones; SG: proteins involved in cell signalling; PT: proteases and their inhibitors; GE: proteins participating in gene expression and its regulation; MS: miscellaneous proteins.

Generally, there were only minor differences between leaf proteomes of hybrids and their parents under control conditions (Fig 7). The largest *number of differentially expressed proteins* was identified between the F1 hybrid CE704×2023 and its paternal parental line 2023 (45 proteins with at least two-fold difference), followed by the 2023×CE704 hybrid *vs* the CE704 inbred line (37 proteins with at least two-fold difference). These proteins belonged to various functional categories; however, the only category that was usually represented only in the control plants was the “Dehydrins” category (Fig 7).

More hybrid-inbred differences in leaf proteome occurred after drought stress, particularly in genotype 2023×CE704 which was characterized by diminished levels of an extremely large number of proteins compared with both parental lines (136, resp. 146, compared to the 2023 and CE704 inbreds, respectively) (Fig 7). Most of the down-regulated proteins (104, *i*.*e*. more than 70%) were shared in both comparisons. Moreover, they highly correlated with proteins that were down-regulated in the 2023×CE704 genotype due to drought stress conditions (S1 Table). Proteins associated with photosynthetic electron-transport chain, carbon fixation and saccharide metabolism were particularly represented, together with those participating in gene expression and its regulation, membrane proteins involved in transport, detoxification proteins, chaperones and proteins involved in amino acid metabolism (Fig 7). However, the CE704×2023 hybrid almost did not differ from its maternal parent CE704 under stress conditions (we found only 20 differentially expressed proteins, 6 belonging to the “Chaperones” category) but showed higher levels of almost 50 proteins (particularly the photosynthetic ones) compared with its paternal parent 2023. Interestingly, most of the up-regulated proteins (30, *i*.*e*., more than 60%) were also over-represented in the reciprocal F1 hybrid 2023×CE704 compared to its paternal parent CE704 under stress conditions and correlated with proteins that were down-regulated by drought in CE704 (S1 Table).

The following tables show the actual *levels* of five proteins with levels that differed *most strongly* between the 2023×CE704 or CE704×2023 F1 hybrids and their maternal or paternal parents. Generally, uniparental heterosis (either negative or positive) for protein levels was not particularly evident in control plants (Tables 4 and 5). The differences between F1 hybrids and their respective parents were not high (mostly 2- to 6-fold), with the exceptions of the mitochondrial glycine-rich RNA-binding protein 2, one protein belonging to ABA-stress-ripening (Asr) inducible protein category and the PsaL subunit of PSI. The inbred line CE704 was characterized by very low levels of these proteins (extremely so in the case of the glycine-rich protein 2) compared with both hybrids (as well as to 2023). Similarly, the leaves of control plants of the 2023 inbred line showed a low amount of the glycine-rich protein 1 (Table 4). Dehydrins were the only group in which heterosis (positive) was consistently higher in the control plants in comparison to the stressed ones, particularly for the CE704×2023 genotype (S1 Table).

**Table 4:** Five proteins that showed the highest positive uniparental heterosis in maize F1 hybrids 2023×CE704 or CE704×2023 under conditions of normal water supply.

Protein	Matching sequence (EST/protein)	23×04/23	23×04/04	04×23/23	04×23/04
**Ranked according to the difference between 2023×CE704 and 2023**					
Glycine-rich protein1	gi|219888685	**10,1**	-1,7	9,9	-1,8
Peroxidase	gi|257738102	**4,9**	-2,1	5,0	-1,9
Protein binding protein	gi|238011090	**3,3**	4,1	1,7	2,1
Peroxiredoxin bcp	gi|226507110	**3,3**	1,0	2,9	-1,1
Hypothetical protein	gi|195655323	**3,2**	-2,3	4,4	-1,9
**Ranked according to the difference between 2023×CE704 and CE704**					
Glycine-rich RNA-binding protein 2	gi|257743787	-1,8	**48,8**	-1,9	53,0
ABA-stress-ripening inducible-like protein	gi|269913328	-1,2	**11,0**	-1,6	9,4
PSI subunit—PsaL	gi|195613284	-2,1	**7,8**	-2,4	7,2
Phosphoenolpyruvate carboxylase	gi|27764449	1,1	**5,2**	-3,7	1,3
Dehydrin 13	gi|195625830	2,6	**4,4**	5,2	6,1
**Ranked according to the difference between CE704×2023 and 2023**					
Glycine-rich protein1	gi|219888685	10,1	-1,7	**9,9**	-1,8
Dehydrin 13	gi|195625830	2,6	4,4	**5,2**	6,1
Peroxidase	gi|257738102	4,9	-2,1	**5,0**	-1,9
Hypothetical protein	gi|195655323	3,2	-2,3	**4,4**	-1,9
Pro-resilin	gi|195620516	2,2	-1,7	**4,2**	1,5
**Ranked according to the difference between CE704×2023 and CE704**					
Glycine-rich RNA-binding protein 2	gi|257743787	-1,8	48,8	-1,9	**53,0**
ABA-stress-ripening inducible-like protein	gi|269913328	-1,2	11,0	-1,6	**9,4**
PSI subunit—PsaL	gi|195613284	-2,1	7,8	-2,4	**7,2**
Dehydrin 13	gi|195625830	2,6	4,4	5,2	**6,1**
Retrotransposon protein	gi|226510042	-1,5	1,6	1,5	**3,0**

The numbers in the individual columns represent the n-fold difference in the protein level between the respective F1 hybrid and its parental inbred line, as derived from the iTRAQ analysis ratios. These ratios are shown as inverse values with a negative sign in case of the higher protein level in the respective inbred line. 23×04 represents the 2023×CE704, 04×23 represents the CE704×2023, 23 represents the 2023 and 04 represents the CE704.

**Table 5:** Five proteins that showed the highest negative uniparental heterosis in maize F1 hybrids 2023×CE704 or CE704×2023 under conditions of normal water supply.

Protein	Matching sequence (EST/protein)	23×04/23	23×04/04	04×23/23	04×23/04
**Ranked according to the difference between 2023×CE704 and 2023**					
Heat shock protein (17.4 kDa, class I)	gi|296512733	**-5,5**	-5,4	-1,3	-1,1
Rubisco LSU-binding protein subunit alpha (GroEL)	gi|257734906	**-2,3**	1,1	-2,4	1,1
PSI subunit—PsaL	gi|195613284	**-2,1**	7,8	-2,4	7,2
Actin-7	gi|238011086	**-2,0**	-4,5	1,5	-1,6
Heat shock protein (70 kDa, 4-like)	gi|308081377	**-2,0**	1,2	-1,0	2,2
**Ranked according to the difference between 2023×CE704 and CE704**					
Heat shock protein (17.4 kDa, class I)	gi|296512733	-5,5	**-5,4**	-1,3	-1,1
Actin-7	gi|238011086	-2,0	**-4,5**	1,5	-1,6
Plastid-lipid-associated protein 2 (PAP/fibrillin family)	gi|226498852	-1,9	**-2,9**	1,2	-1,4
Peroxidase 54	gi|25811927	-1,1	**-2,7**	1,9	-1,1
Glucose-6-phosphate isomerase	gi|293333684	-1,1	**-2,5**	1,3	-1,6
**Ranked according to the difference between CE704×2023 and 2023**					
Phospho*enol*pyruvate carboxylase	gi|27764449	1,1	5,2	**-3,7**	1,3
Chlorophyll *a/b* binding protein (6A)	gi|226503327	1,2	2,4	**-3,5**	-1,8
Late embryogenesis abundant protein, group 3	gi|7387829	1,6	1,9	**-3,2**	-2,8
Chlorophyll *a/b* binding protein (CP24)	gi|226531392	1,1	2,2	**-3,0**	-1,5
6-phosphogluconolactonase	gi|226493090	-1,3	1,1	**-2,6**	-1,8
**Ranked according to the difference between CE704×2023 and CE704**					
Sucrose synthase 3	gi|22121990	1,7	-1,8	-1,0	**-4,1**
Late embryogenesis abundant protein, group 3	gi|7387829	1,6	1,9	-3,2	**-2,8**
Fructose-bisphosphate aldolase (cytoplasmic isozyme)	gi|194703646	-1,0	-1,4	-1,9	**-2,7**
Catalase 3 isoform 1	gi|257675731	-1,0	-1,8	-1,5	**-2,4**
ATPase 2 isoform 1 (plasma membrane)	gi|219888401	-1,1	-1,5	-1,6	**-2,2**

The numbers in the individual columns represent the n-fold difference in the protein level between the respective F1 hybrid and its parental inbred line, as derived from the iTRAQ analysis ratios. These ratios are shown as inverse values with a negative sign in case of the higher protein level in the respective inbred line. 23×04 represents the 2023×CE704, 04×23 represents the CE704×2023, 23 represents the 2023 and 04 represents the CE704.

After 10 days of drought, the differences between F1 hybrids and their inbred parents became more pronounced. Both F1 hybrids retained their positive uniparental heterosis for glycine-rich RNA-binding protein 2, Asr-inducible protein and the PsaL subunit compared with CE704 and showed positive heterosis for some proteins of photosynthetic carbon fixation and saccharide metabolism compared with 2023 (Tables 6 and 7). Extremely high negative heterosis was observed for levels of the chloroplastic ribosomal protein S4 when the F1 hybrid 2023×CE704 was compared with either of its parental lines (Table 7). This phenomenon was caused by a dramatic decrease in the levels of this protein due to drought conditions in the F1 hybrid. Proteins of the photosynthetic electron-transport chain and ATP synthases, as well as some others, showed mostly negative 2023×CE704/2023 a 2023×CE704/CE704 ratios in the stressed plants; again, this was usually caused by a larger decline in their levels in the 2023×CE704 genotype. This change was particularly pronounced for two subunits of PSII (PsbA and PsbE), but it was also evident for some other components of the photosynthetic electron-transport chain (Table 7, S1 Table). On the other hand, the levels of proteins in leaves of the CE704×2023 hybrid subjected to drought did show only rather low negative heterosis compared with its maternal or paternal parent (Table 7, S1 Table).

**Table 6:** Five proteins that showed the highest positive uniparental heterosis in maize F1 hybrids 2023×CE704 or CE704×2023 after 10 days of drought.

Protein	Matching sequence (EST/protein)	23×04/23	23×04/04	04×23/23	04×23/04
**Ranked according to the difference between 2023×CE704 and 2023**					
Calvin cycle protein CP12-1	gi|226493683	**18,4**	2,3	9,3	-1,9
Triosephosphate isomerase, cytosolic	gi|195658525	**5,0**	-3,2	6,1	-2,2
PSII subunit—PsbO	gi|224028817	**4,8**	1,3	4,7	1,1
Pro-resilin	gi|195620516	**4,4**	1,2	3,7	-1,1
GroEL-like type I chaperonin	gi|257720020	**4,1**	-1,7	5,6	-1,2
**Ranked according to the difference between 2023×CE704 and CE704**					
Glycine-rich RNA-binding protein 2	gi|257743787	-2,9	**31,6**	-1,5	40,6
ABA-stress-ripening inducible-like protein	gi|269913328	-1,6	**26,8**	-1,9	22,5
PSI subunit—PsaL	gi|195613284	-1,9	**10,3**	-2,4	8,1
Dehydrin 13	gi|195625830	-1,2	**4,6**	-1,9	3,2
Late embryogenesis abundant protein, group 3	gi|7387829	1,1	**4,0**	-2,1	1,6
**Ranked according to the difference between CE704×2023 and 2023**					
Glycine-rich protein1	gi|219888685	2,2	-8,4	**9,7**	-2,5
Calvin cycle protein CP12-1	gi|226493683	18,4	2,3	**9,3**	-1,9
Pyruvate,*ortho*phosphate dikinase	gi|257659143	3,7	-2,3	**9,2**	-1,0
ATP synthase β subunit (chloroplastic)	gi|902229	1,1	-5,2	**7,4**	1,1
Glyceraldehyde-3-phosphate dehydrogenase B	gi|238011684	3,2	-3,1	**6,8**	-1,6
**Ranked according to the difference between CE704×2023 and CE704**					
Glycine-rich RNA-binding protein 2	gi|257743787	-2,9	31,6	-1,5	**40,6**
ABA-stress-ripening inducible-like protein	gi|269913328	-1,6	26,8	-1,9	**22,5**
PSI subunit—PsaL	gi|195613284	-1,9	10,3	-2,4	**8,1**
Dehydrin 13	gi|195625830	-1,2	4,6	-1,9	**3,2**
Heat shock protein (16.9 kDa, class I)	gi|296512550	1,1	3,7	-1,2	**2,7**

The numbers in the individual columns represent the n-fold difference in the protein level between the respective F1 hybrid and its parental inbred line, as derived from the iTRAQ analysis ratios. These ratios are shown as inverse values with a negative sign in case of the higher protein level in the respective inbred line. 23×04 represents the 2023×CE704, 04×23 represents the CE704×2023, 23 represents the 2023 and 04 represents the CE704.

**Table 7:** Five proteins that showed the highest negative uniparental heterosis in maize F1 hybrids 2023×CE704 or CE704×2023 after 10 days of drought.

Protein	Matching sequence (EST/protein)	23×04/23	23×04/04	04×23/23	04×23/04
**Ranked according to the difference between 2023×CE704 and 2023**					
30S ribosomal protein S4 (chloroplastic)	gi|902224	**-34,7**	-39,4	1,0	-1,2
PSII subunit—PsbE (cytochrome *b*_*559*_ α)	gi|902238	**-21,3**	-23,8	-1,0	-1,3
Histone H4	gi|195635409	**-16,2**	-16,3	-1,3	-1,3
PSII subunit—PsbA (D1 protein)	gi|902201	**-14,9**	-26,0	1,5	-1,4
Carbonyl reductase 3	gi|223948409	**-11,0**	-6,1	-1,0	1,8
**Ranked according to the difference between 2023×CE704 and CE704**					
30S ribosomal protein S4 (chloroplastic)	gi|902224	-34,7	**-39,4**	1,0	-1,2
PSII subunit—PsbA (D1 protein)	gi|902201	-14,9	**-26,0**	1,5	-1,4
PSII subunit—PsbE (cytochrome *b*_*559*_ α)	gi|902238	-21,3	**-23,8**	-1,0	-1,3
Histone H4	gi|195635409	-16,2	**-16,3**	-1,3	-1,3
Glutathione S-transferase	gi|257737838	-6,9	**-12,4**	1,2	-1,4
**Ranked according to the difference between CE704×2023 and 2023**					
Trypsin inhibitor	gi|3264598	-2,1	1,5	**-2,6**	1,3
Inorganic pyrophosphatase	gi|293336730 ^TE^	-3,3	-2,2	**-2,5**	-1,5
PSI subunit—PsaL	gi|195613284	-1,9	10,3	**-2,4**	8,1
Allene oxide synthase	gi|39980758	-2,6	-1,2	**-2,3**	-1,1
Glutathione S-transferase GSTF2	gi|257737308	-4,4	-2,5	**-2,2**	-1,2
**Ranked according to the difference between CE704×2023 and CE704**					
Glycine-rich protein1	gi|219888685	2,2	-8,4	9,7	**-2,5**
PSI subunit—PsaF	gi|226532407	-1,4	-1,9	-1,7	**-2,3**
Triosephosphate isomerase (cytosolic)	gi|195658525	5,0	-3,2	6,1	**-2,2**
Peroxidase	gi|257738102	1,5	-3,5	2,8	**-2,1**
PSII subunit—PsbH	gi|902250	3,3	-2,6	3,7	**-2,1**

The numbers in the individual columns represent the n-fold difference in the protein level between the respective F1 hybrid and its parental inbred line, as derived from the iTRAQ analysis ratios. These ratios are shown as inverse values with a negative sign in case of the higher protein level in the respective inbred line. 23×04 represents the 2023×CE704, 04×23 represents the CE704×2023, 23 represents the 2023 and 04 represents the CE704. TE: *Triticum aestivum* L.

## Discussion

A comparative analysis of various parameters characterizing plant morphology and growth, water status, photosynthesis, cell damage, antioxidative and osmoprotective systems together with an iTRAQ analysis of leaf proteome enabled us to thoroughly examine complex links between various changes occurring in leaves of drought-stressed maize plants at molecular, cell, organ and whole plant levels. Our study was aimed at dissecting the parent-hybrid relationships to better understand the mechanisms of the heterotic effect and its potential association with stress response. The results clearly showed that the four examined genotypes (drought-resistant and -sensitive parental inbred lines, their reciprocal F1 hybrids) have completely different strategies for coping with limited water availability and that positive heterosis in morphological parameters/biomass production, inherent to maize F1 hybrids, was actually disadvantageous when plants encounter drought conditions.

### CE704: What makes this drought-resistant genotype work?

The main mechanisms underlying plant drought resistance are mostly well-defined, however, these mechanisms usually depend on plant exposure to particular drought conditions. Some can confer drought-resistance under conditions of severe drought but are associated with drought-sensitivity under mild or moderate drought stress and *vice versa* [66]. A good example of this is an early stomatal closure, which is often stated to be a typical trait of drought-resistant genotypes, even though several authors did not find a positive correlation between this trait and good biomass production or yield under drought (*e*.*g*. [67–68]). The type of the response to declining water availability (*e*.*g*., whether the respective genotypes have or do not have a change point in their transpiration response to a changing atmospheric vapour pressure deficit) is evidently also important [69]. In our previous study performed with the same drought-susceptible and -resistant inbred lines of maize, the drought-resistant line CE704 not only maintained open stomata under conditions of mild (6 days without water) drought but even increased its g_S_. The results of our analysis of other physiological parameters together with the leaf proteome then led us to propose that this trait enabled it to intensify its photosynthetic processes. This in turn led to the production of more energy for the synthesis and function of important proteins participating in cell protection/detoxification [56]. This time, we prolonged the period without water to 10 days to induce more severe drought stress. While the CE704 line in these conditions did not actively increase its g_S_ and P_N_, the values of these parameters in stressed plants of this genotype exhibited the same level as those measured in control plants. Thus, this inbred line retained its ability to maintain open stomata and to perform fully functional photosynthesis even under conditions of severe water deficit.

The maintenance of open stomata naturally leads to a higher loss of water, as evidenced by the observed decrease in RWC. However, the CE704 line displayed the lowest reduction of this parameter among all genotypes. It therefore had to have some other compensatory mechanism(s) which would either diminish its water losses or intensify its water uptake. We think that the first possibility is true and is associated with its generally smaller size and a lower shoot/root ratio. During the drought simulation period, the gradual changes of soil water content in the pots our plants were grown in were similar for all examined genotypes, *i*.*e*., the amount of available soil water was the same for both parents and their hybrids. However, the smaller size of the aboveground part of the CE704 genotype would indicate a reduced need to cut down transpiration by stomatal closure. A sufficient intake of CO_2_ allows full utilization of light energy in photosynthesis to support necessary processes in plant cells. Because there is no need to limit proteosynthesis or other metabolic processes, plants can increase the efficiency of protective systems. This finding agreed well with the generally smaller effects of drought observed in this genotype as evidenced by the smaller diminution of its biomass, the reduced decrease in its OP, insignificant changes in proline levels, or reduced cell damage as represented by the relative changes in the MI parameter. It was also supported by the observed changes in protein levels. In contrast to its behaviour under mild water stress conditions [56], an increase in proteosynthesis was not observed in the CE704 line under more severe (longer) drought. Nevertheless, it also did not actively down-regulate the levels of many proteins, unlike the drought-susceptible inbred line 2023 and particularly the F1 hybrid 2023×CE704. It is possible that an enforced synthesis of protective proteins earlier during drought response (as observed in our previous study) helped the CE704 genotype to maintain its metabolism more-or-less active and to restrict the damage to cells at acceptable levels even during later stages of drought stress. It is also possible that its earlier intensification of proteosynthesis led to pre-elevated amounts of various proteins, and thus the eventual decrease in their levels during the prolongation of drought would not be readily evident.

The improved drought resistance of CE704 could be also associated with some other properties of this genotype. This inbred line was probably able to emphasize some parts of photosynthetic processes at the expense of others; minimally, our data from the Chl fluorescence analyses and the measurements of photochemical activities in isolated chloroplasts seem to suggest such an option. Although the function of PSII in the CE704 was limited by drought similarly to the other three genotypes (which could in this particular case be caused, *e*.*g*., by its reduced excitonic connectivity among individual PSII units), its PSI complex was affected less negatively. More importantly, the size of the pool of acceptors at the end of photosynthetic electron-transport chain (NADP, ferredoxin) in this genotype actually increased due to drought, in contrast to the drought-susceptible 2023 line and to both F1 hybrids. Thus, the efficiency of the whole electron-transport chain in drought-stressed CE704 plants ultimately was not as negatively affected as in the other three genotypes. The PSI complex is generally thought to be more resistant to stress, but in the case of low CO_2_ fixation efficiency (which would be associated with closed stomata), the lack of NADP^+^ and/or oxidized ferredoxin can cause an electron delay in F_X_/F_A_/F_B_ centres of the PSI complex. The reduced forms of these centres can react with oxygen to generate reactive oxygen species (ROS) which can damage PSI subunits [70]. Consistently, the CE704 genotype indeed showed a much lower decrease in levels of the main PSI subunits PsaA a PsaB compared with the drought-sensitive line 2023 and the 2023×CE704 hybrid. We could perhaps also speculate that the CE704 could have a more active cyclic electron transport around PSI and thus better maintain the overall efficiency of primary photosynthetic processes; however, this would require additional corroborative data.

Under both control and drought conditions, the inbred line CE704 displayed very low amounts of one protein belonging to the Asr-inducible protein category [71]. Transgenic maize plants overexpressing the *Asr1* gene showed increased leaf biomass production [72], and thus low level of this protein in the CE704 genotype could result in the opposite effects, *i*.*e*., lower leaf biomass. Moreover, overexpression of this transcription factor led to greater senescence of leaves in drought-stressed maize plants [73]; the reduced amounts observed in our inbred line should therefore have an opposite effect. This agrees well with our observations of the development and morphology of this genotype regardless of water supply conditions.

One other distinctive characteristic of the CE704 inbred line stood out among the others: a dramatic increase in the activity of APX in leaves of drought-stressed plants accompanied by an associated reduction in the percentage of reduced ascorbate. APX is a well-known antioxidative enzyme that converts hydrogen peroxide to water (using ascorbate as an electron donor), thus eliminating ROS that would damage various cell components [74]. Its role (particularly its chloroplast form) in promoting plant drought tolerance was observed, *e*.*g*., by [75–76] in maize, [77–79] in wheat, [80] in rice or [81] in cowpea. Its increased activity could therefore add another mechanism by which the CE704 genotype would be able to address the negative effects of drought stress.

Curiously, the activities of the other three antioxidative enzymes examined in our study (GR, SOD, CAT) did not show any significant changes under water-limiting conditions, although APX isoforms are thought to strongly cooperate with GR in the ascorbate-glutathione cycle and detoxify hydrogen peroxide generated by SOD. Oxidized forms of ascorbate generated by APX are again reduced by the dehydroascorbate reductase, which uses glutathione as a reducing agent. GR then regenerates glutathione back to its reduced form. However, both ascorbate and glutathione also participate in many other protective and signalling processes in plant cells, and there is an ample evidence that these compounds are at least partly independent [82–84]. Although some studies dealing with the differences between drought-tolerant and -sensitive genotypes documented similar trends of drought-induced changes jointly for several antioxidative enzymes [77, 79], others [85–86] have described differences among the behaviour of the individual enzymes similarly to our group. This result probably depends on a particular plant species and/or genotype, specific drought conditions, the developmental stage of plants and many other factors. Regardless, the ability to increase an APX activity under drought conditions seems to be an inherent property of our drought-resistant maize CE704 genotype, which probably contributes to its better acclimation to stress conditions.

We thus speculate that the mechanisms of drought-resistance in the CE704 inbred line can be connected to its smaller shoot size and leaf area (possibly related to a low level of the Asr1 transcription factor), resulting in reduced water loss from its leaves. This enables the maintenance of open stomata, which means that photosynthesis remains fully functional. During the early stage of drought response, the sufficient amount of energetically rich compounds generated by photosynthetic processes facilitate an increase in proteosynthesis (perhaps particularly of various components of cell protection/detoxification systems). Later, during a more severe drought, this early acclimation mechanism together with the better properties of PSI typical for this genotype and the increased activity of APX in its leaves to a large extent counteract the negative consequences that are generally attributed to drought stress.

### 2023: Predisposition for efficient photosynthesis becomes a burden when drought strikes

In contrast to the CE704 inbred line, 2023 displayed a greater height, aboveground biomass and leaf area under non-stress conditions, as well as higher values of g_S_ and P_N_. Its high efficiency of photosynthesis could be due to the efficient supply of CO_2_ into its leaves, as well as to some other photosynthetic characteristics typical for this genotype. The control plants of the 2023 inbred line showed larger amounts of chlorophylls and slightly higher levels of phosphoenolpyruvate carboxylase (a key enzyme of C4 photosynthetic carbon fixation) compared with CE704 and CE704×2023. The particularly large size of the pool of electron acceptors at the end of photosynthetic electron-transport chain in this inbred line grown under control conditions could be of even more importance. A dramatic difference in the content of PsaL protein between control plants of 2023 and CE704 inbred lines could also be of some interest. The 2023 genotype showed much higher levels of this PSI subunit compared with the other genotype. PsaL protein plays an important role in so-called state transitions, *i*.*e*., processes that regulate the distribution of excitation energy between PSII and PSI [87]. During state II conditions, the extrinsic light-harvesting antennae of PSII (LHCII) move to PSI and associate with its PsaL/PsaH subunits. Pigments bound to these subunits mediate the transfer of the additional energy to a PSI reaction centre [88]. It is possible that, for these reasons, the efficiency of PSI-associated parts of photosynthetic electron-transport chain could be improved in 2023 plants when they are not exposed to stress conditions; this was also supported, in part, by our Chl fluorescence measurements.

The higher PSI efficiency could perhaps be behind another feature observed in control plants of our 2023 inbred line, *i*.*e*., the lowest activity of SOD among all examined genotypes. SOD enzyme detoxifies superoxide radicals by converting them to hydrogen peroxide, thus decreasing the risk of the formation of more dangerous hydroxyl radicals by the Haber-Weiss reaction [89]. As the site of the greatest production of superoxide in plant cells is the thylakoid PSI complex, a larger pool of electron acceptors after PSI could indicate a lower risk of elevated superoxide production [90]. However, higher activities of other antioxidative enzymes (CAT, APX) detected in leaves of the non-stressed 2023 plants could enable an efficient disposition of hydrogen peroxide that would still be generated by other cell processes, thus contributing to the overall higher performance of this genotype in non-stress conditions compared with CE704. This could result in its larger size and greater biomass production. Vikram et al. [91] recently showed that the introduction of the dwarfing allele *sd1* into rice varieties during the Green Revolution was associated with higher susceptibility to drought; thus, according to these authors, the larger plant size would be advantageous under drought conditions. However, we speculate that in case of our drought-stressed maize, the larger size of 2023 could cause various negative consequences that could result in its elevated susceptibility to this stressor. The greater shoot/root ratio displayed by this genotype probably induced higher water loss from its leaves than could be supplied by roots. Thus, it had to close its stomata much earlier to restrict transpiration, resulting in a decrease in photosynthesis and disabling effective protective mechanisms that are dependent on the products of photosynthetic assimilation, as demonstrated in our previous study [56]. Consequently, significantly higher damage to cell membranes occurred in comparison to the other three genotypes.

The inbred line 2023 also showed the largest decrease in OP under drought conditions, signifying that it either produced more compatible solutes or lost more water than the CE704 and both F1 hybrids. We think that the first hypothesis is more probable, because the water losses in at least the 2023×CE704 hybrid were certainly augmented. Cell osmoprotective systems include proline, other amino acids with osmoprotective effects, quaternary ammonium compounds, soluble sugars or sugar alcohols, and other osmolytes [92]. The accumulation of proline in leaves of 2023 was dramatically higher than in CE704. Proline functions as a general osmoprotectant, antioxidant and stress-signalling molecule and its levels in stressed plants usually increase particularly due to high stress intensities [93]. As both our F1 hybrids also showed a high accumulation of proline in leaves when subjected to drought, this would indicate that the differences in changes in OP among these genotypes and 2023 cannot be attributed to this compound.

One group of proteins, *i*.*e*., dehydrins, is also important for an adjustment of cell osmotic pressure, as well as the maintenance of membrane protein stability and protection of various cell macromolecules against degradation [94–95]. Their accumulation is usually considered to be a general symptom of drought stress [96]. Heat shock proteins (HSPs) functioning as molecular chaperones bind to denatured substrates and regulate their folding, thus affecting their degradation, accumulation as well as localization; a group of small HSPs also prevents irreversible protein aggregation [97]. Their association with drought tolerance seems to be rather ambiguous [98–109]. We observed the accumulation of dehydrins and molecular chaperones in all four genotypes examined; however, the 2023 inbred line showed a particularly high accumulation of these proteins.

The effectivity of at least some reactions of cell antioxidative system remained the same in leaves of drought-stressed plants of this genotype as in control plants, as indicated by the unchanged activities of the four antioxidative enzymes examined as well as levels of most proteins belonging to the “Detoxification” category that we detected in our proteomic analysis. However, this finding does not provide information about the adequacy/inadequacy of antioxidative protection and may even suggest that it was insufficient. Increased ROS production is generally accepted as a typical stress response and would certainly require a more efficient detoxifying system to induce stress tolerance. This should be reflected in the degree of damage to cell components, particularly in chloroplasts in which large amounts of ROS are generated. The damage to cell membranes in the 2023 genotype was evident in the higher value of MI and the damage to chloroplast proteins was demonstrated by the results of the proteomic analysis. The drought-stressed plants of the 2023 inbred line were characterized by a rather prominent down-regulation of several proteins of the photosynthetic electron-transport chain and chloroplast ATP synthesis. The 10 kDa phosphoprotein of PSII (PsbH) showed a particularly large decrease under stress conditions. The PsbH protein plays a role either in the assembly or stability of PSII complex and can also participate in the repair of photodamaged PSII under high irradiance conditions [110–111]. Its reduced levels in the 2023 inbred line could contribute to the diminished functionality of photosynthetic electron-transport chain as suggested by the measurements performed with isolated chloroplasts as well as by the Chl fluorescence measurements.

The levels of the PsbO subunit of the PSII OEC decreased in drought-stressed leaves of 2023 more than in the other three genotypes. Various authors have described a decrease in the levels of this protein in the leaves of some drought-sensitive genotypes but an increase, no changes or at least a more gradual decrease in a corresponding drought-resistant genotype [98–99, 104, 107, 112–114]. However, others did not find genotypic differences in the drought-induced changes in this OEC component [101, 115–116]. In any case, our inbred line 2023 showed the smallest inactivation of the OEC due to drought among all four examined genotypes, so any problems caused by the reduced amount of this protein were probably not particularly significant. This genotype was also characterized by the least impairment of excitonic connectivity between PSII complexes, and thus this feature of PSII functionality did not contribute to the diminished functionality of its photosynthetic electron-transport chain.

On the other hand, the changes observed in some PSI-associated features were rather prominent. The reduced size of the pool of PSI end electron acceptors was most distinctive in this inbred line. The 2023 also differed from the other three genotypes in the levels of ferredoxin-NADP reductase in leaves of drought-stressed plants. This could indicate that the down-regulation of this final portion of the photosynthetic electron-transport chain was responsible for the decreased efficiency of primary photosynthetic processes in the drought-stressed 2023 plants to a greater extent than the PSII-associated parts. Most authors who examined genotypic differences and presented data from leaf proteomic analyses of drought-stressed plants also encountered more (or earlier) decreased levels of ferredoxin-NADP reductase in their drought-sensitive genotypes in comparison to drought-tolerant ones [98–99, 106, 116–117]. Faghani *et al*. [118] reported an increase in the levels of ferredoxin-NADP reductase in the leaves of the drought-stressed wheat cultivar with higher resistance to this stressor but not in leaves in the drought-sensitive cultivar. Li *et al*. [109] stated that *Stipa purpurea* plants originating from more arid areas and thus more resistant to drought had higher levels of this protein even under non-stress conditions in comparison to plants with a worse adaptive ability to drought. However, some other authors did not find any particularly significant differences between genotypes with different susceptibilities to drought in the quantitative response of this protein [101, 114, 119] or even observed an increase in its levels in a drought-sensitive genotype but not in a drought-resistant one [105]. Again, the variability in experimental design and examined plant species probably underlies this evident discrepancy in published results.

Subunits of chloroplastic ATP synthase belong to another group of proteins associated with the efficiency of primary photosynthetic processes and down-regulated in the 2023 inbred line. Data from proteomic studies dealing with differences between drought-sensitive and -tolerant genotypes vary. Some authors describe a drought-induced decrease in the levels of β subunit of this complex in a genotype with a higher susceptibility to drought but an increase or at least no changes in a genotype showing a better adaptability to this stressor [98, 102–103, 120]. A similar situation was observed for an α subunit in this complex [96]. Li *et al*. [109] stated that *Stipa purpurea* plants adapted to drought conditions had greater amounts of the δ subunit of ATP synthase and, when exposed to drought, were able to increase levels of β, γ and ε subunits whereas plants that were more susceptible to drought increased only levels of the β subunit. In leaves of another grass species, *Cynodon dactylon*, the level of α subunit was induced by drought in a drought-tolerant cultivar but not in a drought-susceptible one [105]. Faghani *et al*. [118] reported decreased levels of the γ subunit of ATP synthase in a drought-sensitive cultivar of wheat whereas the amounts of this protein in leaves of drought-tolerant plants did not change. However, other authors [99, 114, 121] observed a down-regulation of the α subunit in leaves of both drought-tolerant and -sensitive genotypes of French bean, eucalyptus or sugarcane plants. Cheng *et al*. [101] described the same situation for a β subunit in leaves of drought-stressed wheat and Jangpromma *et al*. [122] observed a similar increase in leaves of drought-tolerant and -sensitive sugarcane plants for α and β subunits. The situation regarding intraspecific differences in the response of various subunits of this complex to drought is thus far from being clear, although it seems that with an increasing susceptibility to drought a more negative response is generally observed. This is consistent with our results.

In addition to proteins of the photosynthetic electron transport and chloroplastic ATP synthase, the 2023 inbred line was also characterized by very distinctive drought-induced changes in proteins associated with photosynthetic carbon fixation. Chloroplastic CP12 protein, GAPDH B and PPDK were among the five most highly down-regulated proteins in this genotype. Some other proteins participating in the carbon fixation (*e*.*g*., small subunit of ribulose-1,5,bisphosphate carboxylase/oxygenase, chloroplastic phosphoglycerate kinase, transketolase, sedoheptulose-1,7-bisphosphatase) were also negatively affected in the 2023 inbred line much more than in the other three genotypes. Thus, down-regulation of this group of proteins under drought conditions must be regarded as an inherent attribute of this inbred line and is likely connected to decreased uptake of CO_2_.

CP12 is a small chloroplastic protein that is sensitive to redox conditions, which regulates (*via* thioredoxin) the enzymes of Calvin-Benson cycle. The formation of its complex with GAPDH/phosphoribulokinase leads to the protection of these enzymes against oxidative stress [123]. In *Chlamydomonas reinhardtii*, it specifically serves as a chaperone that protects GAPDH against heat-induced aggregation and inactivation [124]. The observed changes in the levels of this protein could thus result in a greater stress susceptibility of GAPDH. GAPDH catalyses the conversion of 1,3-*bis*phosphoglycerate to glyceraldehyde-3-phosphate. Similarly to our group, most other authors found that the levels of both chloroplastic forms of this protein (either A or B isozymes) decrease due to drought stress more (or earlier) in drought-sensitive genotypes of various plant species than in drought-tolerant ones [96, 98, 106, 112, 116–117]. However, an opposite situation was described by [125], who worked with drought-tolerant and -sensitive genotypes of sorghum. Katam *et al*. [104] reported an increased level of the A isozyme of chloroplastic GAPDH in drought-stressed plants of groundnut for a tolerant genotype but an absence of this form in leaves of a sensitive genotype, which means that there can be at least interspecific differences in this respect.

PPDK catalyses the phospho*enol*pyruvate regeneration phase of the C4 carbon fixation pathway. The decreased level of PPDK can thus be associated with the diminished CO_2_ fixation caused by decreased g_S_ in the 2023 line under water-limiting conditions. Doubnerová-Hýsková *et al*. [126] presented evidence that in drought-stressed tobacco (which is, however, a C3 plant), its levels increased. They later suggested that this enzyme could, together with other enzymes of the C4 cycle, be involved in the conversion of NADP to NADPH [127]. Because NADPH is an important component of various cell antioxidative and osmoprotective mechanisms, the reduced levels of PPDK in our 2023 inbred line of maize could perhaps also contribute to its susceptibility to drought. Intraspecific differences in the levels of this enzyme are reported less frequently in comparison to other enzymes of photosynthetic carbon fixation and saccharide metabolism. Ji *et al*. described the down-regulation of this protein in a sensitive genotype of rice but not in a drought-tolerant genotype [120]. Jedmowski *et al*. [125] reported a drought-induced elevation in the amounts of this protein for drought-tolerant sorghum plants but no changes in leaves of drought-sensitive plants. Finally, Wang *et al*. [102] found that levels of PPDK decrease in leaves of barley susceptible to drought and increase in another cultivar with better drought resistance but only after drought simulation ended and the plants were recovering from stress. Similarly to our group, these reports indicate that lower levels/drought-induced decrease is associated with a greater plant susceptibility to water limiting conditions.

Based on all the above mentioned findings, the following picture emerges: the 2023 inbred line is genetically predisposed (probably due to its more efficient photosynthetic processes) to a larger size. However, when it encounters drought conditions, its higher shoot/root ratio induces an early stomatal closure, which together with an unchanged efficiency of its detoxifying systems creates various negative effects particularly on proteins of photosynthetic carbon fixation. Coupled to the reduced amounts of PSI electron acceptors, the destabilization of PSII (probably caused mostly by the diminished levels of its PsbH protein) and the insufficient supply of CO_2_ due to stomatal closure, this leads to an overall reduction of photosynthetic efficiency and, consequently, to an insufficient energy supply for various metabolic processes. The increased accumulation of osmoprotectants, which is characteristic for this genotype, is not sufficient to overcome this problem. It can be regarded rather as a manifestation of the stress sensitivity of this genotype. The overall negative response of 2023 to drought stress was also evidenced by the high degree of membrane injury as well as by the effects on its general morphology, growth and development.

### 2023×CE704: Large size confers a distinct disadvantage in drought: The case of diminished proteosynthesis

The 2023×CE704 genotype displayed positive heterosis in morphological parameters that is typical for a majority of maize F1 hybrids. It was characterized by a rather high g_S_ under non-stress conditions, similarly to its maternal inbred line. However, although this enabled efficient photosynthesis, it was also associated with a high transpiration rate, perhaps augmented by its much larger leaves. Consequently, control plants of this F1 hybrid displayed a lower WUE in comparison to both its parents. When subjected to drought, this predisposition to high transpiration, together with its extremely large leaf area, low shoot/root ratio and the observation that 2023×CE704 slowed its development to a lesser extent than its maternal line (which developed fewer leaves under drought conditions), created a distinct disadvantage for this hybrid. Even the closure of stomata did not help with the loss of water from its leaves, which was greatest among all examined genotypes. In contrast, closed stomata indicated an insufficient supply of CO_2_ for photosynthesis, preventing the production of the energetically rich compounds necessary for various metabolic processes, in turn inducing rather dramatic changes in the amounts of many leaf proteins.

Although the levels of some proteins (dehydrins, HSPs) in leaves of the 2023×CE704 hybrid increased, a much greater number of proteins were down-regulated. The 2023 was also characterized by the down-regulation of some proteins; however, while this decrease in the 2023 genotype involved mostly proteins of photosynthetic carbon fixation, thylakoid electron-transport chain and chloroplast ATP synthesis, in the F1 hybrid 2023×CE704 a more general phenomenon was observed involving various other classes of proteins. This was probably associated more with an impaired proteosynthesis than with an increased protein degradation, as we found reduced levels of many ribosomal proteins. Indeed, the proteolytic subunit of ATP-dependent Clp protease was one of the most strongly down-regulated proteins in 2023×CE704. However, this could actually further contribute to a greater sensitivity of this genotype to drought because it would indicate that damaged, denatured or incorrectly folded proteins would not be as efficiently degraded. Cheng *et al*., who examined changes in the leaf proteome in wheat subjected to drought hypothesized that a greater efficiency of proteolytic processes could be a basis for a reduced susceptibility to this stressor [107].

Literature data on the changes in ribosomal proteins due to drought are not entirely clear; it is evident that different proteins of both large and small ribosomal subunits can respond differently. The characteristics of these changes probably also depend on particular conditions of drought stress (intensity/length, type of simulation), on plant species and (as seen in our case) on genotype. Li *et al*. found that plants of *Stipa purpurea* that were adapted to a more arid environment were able to increase levels of various subunits of both cytosolic and chloroplast ribosomes in response to drought but this phenomenon was not observed in a more sensitive variant of the same species [109]. Another study dealing with intraspecific differences in wheat subjected to drought conditions documented a decrease in one protein of the 60S ribosomal subunit in a sensitive cultivar but not in a tolerant one [118]. Regarding chloroplast ribosomes, some authors have observed an increase in some proteins of both large and small ribosomal subunits to be associated with a better drought tolerance [96, 99, 101, 107], although this was not true for all proteins belonging to this category [96, 107].

In our case, the reduction of ribosomal proteins observed in the 2023×CE704 hybrid applied to some subunits of cytoplasmic ribosomes but was much more evident for the components of chloroplast ribosomes. This finding is very interesting and suggests the possibility of strongly limited proteosynthesis particularly in the chloroplasts of this genotype. As the main type of proteins synthesized in these organelles are components of photosynthetic thylakoid complexes, it would be expected that this category of proteins would generally display the greatest reduction of levels. This was exactly the case; indeed, in the 2023×CE704 hybrid, the PsbA (D1) and PsbE (cyt_*b559*_ α) subunits of PSII were among the strongest down-regulated proteins and genes for both proteins are located in the plastid genome. The PsbA subunit constitutes one of the main parts of the core of the PSII reaction centre, binding (together with its associated PsbD) all necessary cofactors for photosynthetic electron transport [128]. The levels of PsbD protein were also rather prominently reduced in the drought-stressed plants of our F1 hybrid, although not to extent as great as the PsbA levels. This was probably caused by the particular susceptibility of PsbA to photodamage associated with various stresses [129].

The PsbE subunit is also necessary for a fully functional PSII reaction centre even though it does not participate in the linear electron transport from water to plastoquinone. Its haem group belongs to co-factors of the secondary electron transport pathways in PSII and enables a cyclic electron transport around this complex. These pathways should protect PSII against damage induced when the reduction of the primary PSII donor with an electron from the OEC is prevented [130]. The decreased levels of this protein should thus cause the PSII in leaves of the 2023×CE704 hybrid to become particularly susceptible to inhibition, which agrees well with our functional measurements of the activity of this complex. The 2023×CE704 genotype also showed the largest decrease in excitonic connectivity between PSII units which would further diminish the efficiency of primary photosynthetic processes. Another protein of PSII with rather significantly drought-reduced levels in 2023×CE704 was CP47 (PsbB), which is a component of the inner light-harvesting antennae of PSII with a large lumenal loop that makes of part of the OEC [131].

Our 2023×CE704 plants also displayed a down-regulation of the PsbQ subunit of the OEC. The role of this protein in PSII remains mostly unclear [132]. It probably stabilizes PsbP, another OEC protein, which in turn enables the binding of the essential OEC cofactors Ca^2+^ and Cl^-^ and thus the function of this complex [133]. Additionally, it may be required for efficient formation of the supercomplex of PSII with proteins of its external LHC antennae [134]. The data on drought-induced changes in this protein coming proteomic analyses conducted in drought-tolerant and -sensitive genotypes of various plant species are very ambiguous. Kolenc *et al*. [116] reported a greater decrease in this protein in leaves of hop plants that were more susceptible to drought than in drought-tolerant ones. Ford *et al*. [113] found decreased levels of PsbP in a tolerant genotype of wheat but not in a sensitive genotype. Hao *et al*. [100], who analysed the same species, stated that this protein is up-regulated by drought and that this up-regulation is slightly increased in a tolerant cultivar than in a sensitive one.

In any case, our 2023×CE704 plants showed particularly strong inactivation of the OEC due to drought as suggested by the fluorescence measurements, which agrees well with the changes observed at the protein level. However, PSII was not the only complex of chloroplast thylakoid membranes with subunits that were significantly reduced in leaves of drought-stressed plants of 2023×CE704. Similarly to 2023, various subunits of chloroplast ATP synthase were negatively affected in this genotype. Some LHC proteins, subunits of PSI as well as two proteins of the cytochrome *b*_*6*_*f* complex also showed a drought-induced decrease to a greater extent in 2023×CE704 than in the other three examined genotypes. Surprisingly, components of PSII other than members of its OEC usually do not show any distinctive changes in drought-stressed plants. Only Zhou *et al*., who analysed the leaf proteome of *Malus domestica*, described such a response for various PSII components and identified these proteins in more detail. According to the data they presented, both increased, decreased or no changes in their levels seemingly lacked any clear relationship to drought susceptibility/resistance; however, they stated that an increase in the levels of PSII (and PSI) subunits was generally higher in a drought-tolerant cultivar compared to a drought-sensitive one [103].

To summarize, the highly negative response of the 2023×CE704 to drought is fundamentally associated with the large aboveground biomass of this F1 hybrid. Although at first glance this response appears to be similar to that of its maternal parent 2023, the intrinsic changes that occur in leaves of drought-stressed plants of these two genotypes are different. Being predisposed to high transpiration and less efficient photosynthesis under non-stress conditions, when drought occurs, the 2023×CE704 hybrid experiences high water losses that cannot be overcome even by early stomatal closure. This is due to its greater number of large leaves and the generally low shoot/root ratio. After stomatal closure, the 2023×CE704 hybrid suffers from insufficient production of the energetically rich compounds necessary for biosynthetic processes. Consequently, its proteosynthesis (especially in chloroplasts) is strongly reduced and dramatic reduction of the amounts of proteins that participate in primary photosynthetic processes occurs (particularly PSII components). This, together with a high inefficiency of its OEC and a bad functional connectivity between individual PSII complexes, further decreases the amount of energy potentially utilizable by primary photosynthetic processes, amplifies the damage to various cell components and further reduces the amounts of various proteins in drought-exposed plants of this F1 hybrid. Osmoprotective compounds induced by drought are not sufficient to protect its cells from injury, particularly because their accumulation is reduced in comparison to the 2023 maternal parent.

### CE704×2023: Something from its mother, something from its father, something of its own

The behaviour of the CE704×2023 hybrid under drought stress was the most interesting one. The changes in its MI suggested that cells of this F1 hybrid are not as greatly damaged as in its paternal parent or its reciprocal sibling. Similarly to these genotypes, CE704×2023 was also characterized by a large height, an aboveground biomass and a leaf area (although less so than 2023×CE704). Its shoot/root ratio was more-or-less the same as in its reciprocal sibling both under control and stress conditions. Leaves of the non-stressed CE704×2023 plants showed a WUE that was similarly low to the 2023×CE704 genotype (compared to both their parents). However, in this case this was not associated with higher transpiration but with a lower P_N_.

The reasons for this lower photosynthetic efficiency are not particularly clear; however, there is one feature of primary photosynthetic processes in which the control plants of the CE704×2023 genotype differed both from its reciprocal sibling and paternal parent. The CE704×2023 hybrid was characterized by lower levels of LHC proteins. Chlorophylls and carotenoids bound to LHC proteins capture the excitonic energy from light and transfer it to PSII and PSI, where it is transformed into energy necessary for electron transport. Smaller LHC antennae under non-stress conditions could reduce the efficiency of energy capture and thus diminish photosynthesis. However, under drought conditions, lower amounts of LHC proteins should rather confer an advantage because the excess excitation energy (not utilizable by primary photochemistry due to an over-reduced electron-transport chain) would not have to be dissipated to as great an extent [135]. Wang *et al*. [102] found that at least some LHC proteins in leaves of two barley genotypes differing in drought susceptibility showed intraspecific differences in their levels even prior to drought: the genotype that was more drought-resistant had higher levels of some LHC subunits. This would perhaps agree with our results, because the CE704×2023 genotype was not particularly resistant to drought. However, although Li *et al*. [109] observed a similar situation for some LHC proteins in *Stipa purpurea* plants originating from arid conditions and thus better adapted to water deficiency, an opposite situation was observed for other LHC proteins.

Changes in the levels of LHC proteins after exposure of plants to drought stress were found in various proteomic studies examining differences between drought-sensitive and -resistant genotypes. However, to date, these data are rather contradictory. Some authors have described an up-regulation of at least some LHC proteins in plants that are resistant to water stress but no changes in susceptible ones (*e*.*g*., [99, 102–103]). Others have confirmed a drought-induced elevation of their levels for drought-tolerant genotypes but observed decreased amounts of these proteins in drought-susceptible genotypes [104], or a decrease in plants showing a higher susceptibility to water deficiency and no changes in plants that are resistant to this stressor [96]. There are also studies reporting a down-regulation of LHC proteins both in drought-resistant and sensitive genotypes [112, 114] or an increase of levels of some LHC proteins, a decrease of others in a genotype with a lower susceptibility to drought conditions, but no changes in a highly susceptible genotype [119]. After 10 days of drought, we found that either no changes or even an increase in the amounts of some LHC proteins occurred in the CE704×2023 hybrid whereas the inverse situation was observed in the other three genotypes. This phenomenon could contribute to the negative effects of drought on photosynthesis observed in CE704×2023.

Another protein with differing levels among the CE704×2023 hybrid, its reciprocal sibling and both parents prior to drought stress was a late embryogenesis-abundant (LEA) protein belonging to the group 3 of LEA proteins [136]. This protein is thought to be associated with stress tolerance in several plant species [137–140]. Li *et al*. [109], in their previously mentioned study conducted in *Stipa purpurea* subjected to drought reported that plants that were less susceptible to drought showed lower levels of this protein when non-stressed by drought, but under conditions of water deficiency, these plants showed an increase in protein levels. In contrast, plants that were susceptible to drought did not display this effect. Our CE704×2023 genotype was characterized by lower levels of this protein in leaves of control plants in comparison to both parents. However, it showed the highest up-regulation when plants of the CE704×2023 hybrid were exposed to drought, which could perhaps somehow aid this genotype in partially overcoming the negative effects of water deficiency. The inbred line 2023 also showed a strong induction of this protein, so CE704×2023 could inherit this feature from its father; however, beneficial effects of this protein on this inbred line would be overcome by other negative aspects of the 2023 physiology as discussed above.

After 10 days of drought, the CE704×2023 hybrid closed its stomata and inhibited transpiration similarly to the 2023×CE704 and the 2023 genotypes, but the decrease in its leaf RWC was not as high as in the 2023×CE704. This could be due, at least in part, to the lower transpiration rate characterizing this genotype under control conditions. In addition, its OP decreased similarly to the drought-resistant inbred line CE704, *i*.*e*., also not as prominently as in the 2023 or the 2023×CE704 genotypes. This difference could perhaps be caused by a higher retention of water, but this is only a speculation. It certainly was not caused by significant differences in proline accumulation.

However, an inherent characteristic of the CE704×2023 genotype was that its leaves contained somewhat higher amounts of dehydrins compared with both its parents, even under control conditions. Higher dehydrin levels prior to drought could indicate that CE704×2023 could be “better prepared” for subsequent drought and would not need to increase the amounts of these proteins (or adjust its OP) as much as 2023 or 2023×CE704. Consequently, this hybrid imitated the drought-resistant CE704 genotype (its maternal parent), which also did not up-regulate dehydrin levels under drought conditions to such a large extent as the other two examined genotypes, although for different reasons. Some authors also hypothesize that dehydrins could function as molecular chaperones similarly to HSPs, preventing interactions between denatured proteins and their drought-induced aggregation. They could enable a switch to an ordered protein state, *i*.*e*., to induce correct protein folding, further reducing protein degradation [141]. In general, only a very few proteins were down-regulated in the leaves of drought-stressed plants of this genotype, similarly to its maternal parent, *i*.*e*., CE704.

Interestingly, the proteins that *were* down-regulated in the CE704×2023 hybrid belonged almost exclusively to the photosynthetic carbon fixation and saccharide metabolism group. Thus, a strong similarity between the CE704×2023 and its paternal parent in the types of proteins that were decreased under drought conditions was evident. Among the five proteins with the most marked changes in drought-exposed CE704×2023 were the same proteins described for 2023, *i*.*e*., GAPDH and CP12 chloroplastic protein. Moreover, another down-regulated protein in the CE704×2023 genotype was the PsbH subunit of PSII, which displayed similar response to that in the 2023 inbred line. Additionally, the difference in the size of the pool of PSI end electron acceptors between control and stressed plants was greater than in 2023×CE704 and the rate of the PSI reduction decreased the most among all examined genotypes. Thus, this F1 hybrid seemed to inherit from its father some of the worst features of its drought-induced response of components of the photosynthetic apparatus. This could perhaps contribute to the observation that the negative effects of drought on primary photosynthetic processes (both PSII- and PSI-related parts of the thylakoid electron transport) as well as on the net photosynthetic rate were similarly as strong as those observed in 2023×CE704. Even a good excitonic connectivity among PSII units (also similar to that observed in the paternal parent 2023) was not sufficient to ensure efficient photosynthetic electron transport.

We can conclude that the CE704×2023 hybrid displays similarly negative drought-induced effects on its growth as the other two examined genotypes characterized by a large shoot size (2023 and 2023×CE704). These morphological properties are again associated with a need for an early stomatal closure and reduced transpiration. The water loss was not as prominent in this genotype, suggesting that perhaps its lower predisposition to transpiration in combination with a slightly smaller leaf area partly fills the demand for water retention. Higher amounts of dehydrins, which are typical for this genotype even under non-stress conditions, probably partially protect its cells and their components from drought-induced injury and generally assist in preventing an unordered state of other proteins, thus enabling a reduced degree of protein degradation. However, photosynthesis is not well protected in this genotype, mostly due to various paternally inherited negative features of both primary and secondary photosynthetic processes (proteins involved in carbon fixation, PSI-associated parts of photosynthetic electron-transport, instability of PSII). Additional factors that negatively affect photosynthetic efficiency inherent to CE704×2023 are related to its light-harvesting antennae—although originally smaller, the synthesis of the respective proteins does not diminish under drought conditions, resulting in a combination with a more negatively affected photosynthetic electron transport in greater amounts of non-utilizable excitation energy. All these factors reduce the efficient conversion of light energy into a chemical product, in the final result negatively impacting the performance of CE704×2023 under drought.

### Conclusions

A combined analysis of plant morphology, physiology and the leaf proteome in two maize inbred lines and their F1 hybrids clearly showed that an inherent property of F1 hybrids in non-stress conditions, *i*.*e*., positive heterosis in morphological parameters (or, more generally, a larger plant body) becomes a distinct disadvantage when the water supply is limited. However, although the increased loss of photosynthetic efficiency is always in the background of this disadvantage, the precise causes and consequences of the original predisposition for faster growth and biomass accumulation can differ even between reciprocal hybrids. Both maternal and paternal parents can be imitated by their progeny in some aspects of drought responses; nevertheless, other aspects may be quite unique to a particular hybrid. The importance of analysing *both* reciprocal F1 hybrids when examining various physiological and molecular features of heterosis is thus substantially evident. In addition, our study also proved that the strategy of leaving stomata open even when the water supply is limited (coupled with a smaller body size and some other physiological properties) is associated with drought-resistance not only under mild drought but also under more severe drought conditions.

## Supporting information

S1 Table The list of the identified proteins and their classification into functional categories. Sheet “All proteins” shows details to identification and quantification (iTRAQ ratios) of all matching iTRAQ-labelled peptides characterized by tandem MS/MS. Functional categories sheets present only proteins which differed at least two fold in any of the stated ratios (shown in red for negative values and in blue for positive values). Columns with orange background in these sheets show different S/C ratios (or, for proteins whose levels decreased in the stressed plants, –1/[S/C] ratios) characterizing the differences between stressed (S) and control (C) plants of two maize inbred lines 2023 (23) and CE704 (04) and their F1 hybrids 2023×CE704 (23×04) and CE704×2023 (04×23). Columns with green background show different C_F1_/C_P_ ratios characterizing the differences between the respective F1 hybrids and their inbred parental lines under control conditions (in case of the higher protein level in inbred line these ratios were expressed as –1/[C_F1_/C_P_)]), columns with yellow background show similar comparisons for plants under stress conditions (S_F1_/S_P_, resp. –1/[S_F1_/S_P_]).
(XLSX)

S1 File Changes in the volumetric soil water content during 10 days of drought simulation. The means ± SD (n = 10) are shown and the statistical significancy of the differences between the respective variants (*i*.*e*., between the control and stressed plants of each individual genotype or between individual genotypes under control *vs* stress conditions) as determined for each day separately by the Games-Howell test is given below the graph (* … significant at p < 0.05, ns … not significant). C … control, S … drought stress.
(PDF)

S2 File Changes in the gas exchange parameters during 10 days of drought simulation. The stomatal conductance (g_S_), the net transpiration rate (E) and the net photosynthetic rate (P_N_) were measured in leaves of maize inbred lines 2023 and CE704 and their F1 hybrids 2023×CE704 and CE704×2023. For better clarity, only the means (n = 22–28 plants) are shown in the graphs and the statistical significancy of the differences between the respective variants (*i*.*e*., between the control and stressed plants of each individual genotype or between individual genotypes under control *vs* stress conditions) as determined for each day separately by the Games-Howell test is given below the respective graphs (* … significant at p < 0.05, ns … not significant). C … control, S … drought stress.
(PDF)
