# Supplementary material for: The disadvantages of being a hybrid during drought: A combined analysis of plant morphology, physiology and leaf proteome in maize
Source: PLoS One. 2017 Apr 18;12(4):e0176121. doi: 10.1371/journal.pone.0176121 (PMC5395237; doi:10.1371/journal.pone.0176121)
Supplement: S1 File — The means ± SD (n = 10) are shown and the statistical significancy of the differences between the respective variants (i.e., between the control and stressed plants of each individual genotype or between individual genotypes under control vs stress conditions) as determined for each day separately by the Games-Howell test is given below the graph (* … significant at p < 0.05, ns … not significant). C … control, S … drought stress. (PDF) [file pone.0176121.s002.pdf]

Changes in the volumetric soil water content as measured in pots with maize inbred lines 2023 and CE704 and their F1 hybrids 2023×CE704 and CE704×2023 during 10 days of drought simulation. The means ± SD (n = 10) are shown and the statistical significancy of the differences between the respective variants (*i.e.*, between the control and stressed plants of each individual genotype or between individual genotypes under control *vs* stress conditions) as determined for each day separately by the Games-Howell test is given below the graph (* … significant at $p < 0.05$, ns … not significant). C … control, S … drought stress.

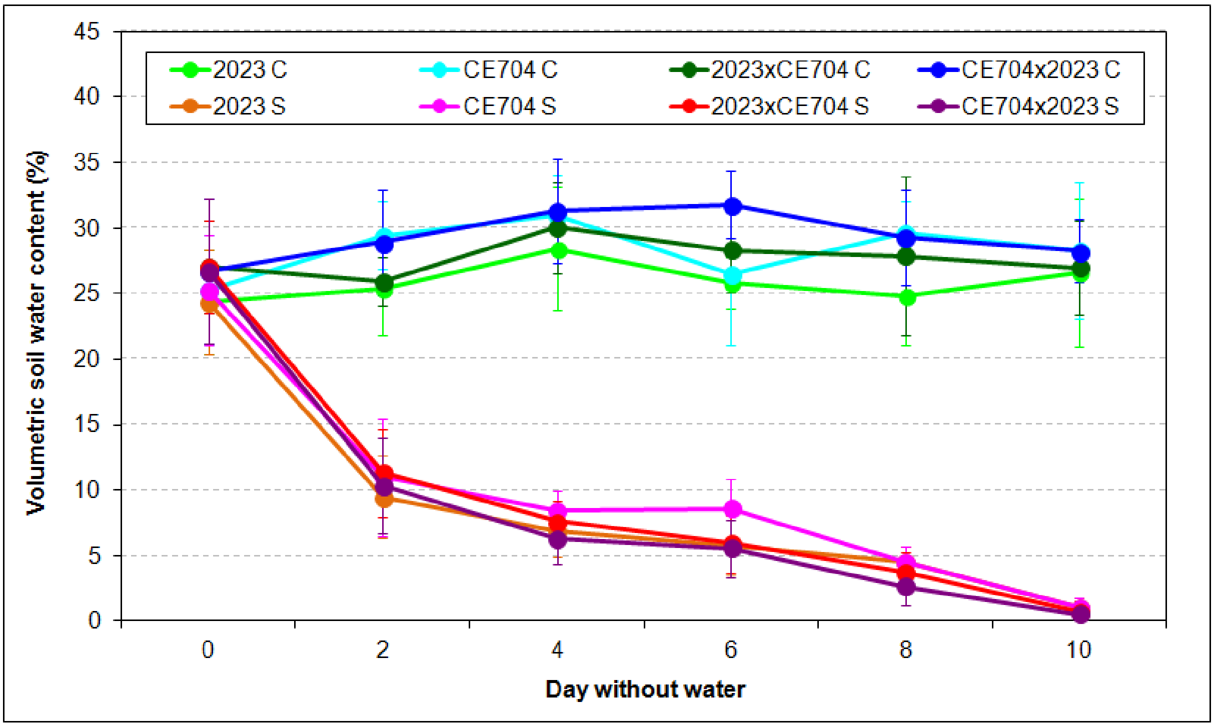

	Day 0	Day 2	Day 4	Day 6	Day 8	Day 10
2023 C – 2023 S		*	*	*	*	*
CE704 C – CE704 S		*	*	*	*	*
2023×CE704 C – 2023×CE704 S		*	*	*	*	*
CE704×2023 C – CE704×2023 S		*	*	*	*	*
2023 C – CE704 C	ns	ns	ns	ns	ns	ns
2023 C – 2023×CE704 C	ns	ns	ns	ns	ns	ns
2023 C – CE704×2023 C	ns	ns	ns	*	ns	ns
CE704 C – 2023×CE704 C	ns	*	ns	ns	ns	ns
CE704 C – CE704×2023 C	ns	ns	ns	ns	ns	ns
2023×CE704 C – CE704×2023 C	ns	ns	ns	ns	ns	ns
2023 S – CE704 S		ns	ns	ns	ns	ns
2023 S – 2023×CE704 S		ns	ns	ns	ns	ns
2023 S – CE704×2023 S		ns	ns	ns	ns	ns
CE704 S – 2023×CE704 S		ns	ns	ns	ns	ns
CE704 S – CE704×2023 S		ns	ns	ns	ns	ns
2023×CE704 S – CE704×2023 S		ns	ns	ns	ns	ns
